# Vitamin U Attenuates Acute Aflatoxin B_1_-Induced Liver Injury in Mice: Biochemical, Histological and Transcriptomic Evidence

**DOI:** 10.3390/vetsci13070621

**Published:** 2026-06-26

**Authors:** Liyu Yang, Jiaxin Liu, Xuanxuan Zhang, Yake Wang, Shufan Liu, Chenxi Ling, Xinfeng Li, Kun Liu, Yong Huo, Guangwei Zhao, Qiuliang Xu, Hongyu Deng, Congcong Li

**Affiliations:** 1 College of Animal Science and Technology, Henan University of Animal Husbandry and Economy, Zhengzhou 450046, China; 2 Henan Pig Bio-Breeding Research Institute, Zhengzhou 450046, China; 3 College of Animal Sciences, Xinjiang Agricultural University, Wulumuqi 830052, China; 4 Henan Key Laboratory of Livestock and Poultry Genetic Improvement and Healthy Breeding, Zhengzhou 450046, China; 5 Henan Livestock and Poultry Genetic Resources Protection Engineering Technology Research Center, Zhengzhou 450046, China

**Keywords:** Aflatoxin B_1_, Vitamin U, mice, acute liver injury, oxidative stress

## Abstract

Aflatoxin B_1_ (AFB_1_) causes acute liver injury in livestock. This study evaluated whether Vitamin U could alleviate AFB_1_-induced hepatotoxicity in mice. AFB_1_ (3 mg/kg) reduced PLT, PCT, and EOS counts, caused hepatic vascular congestion, and decreased GSH-Px activity. Vitamin U (50 mg/kg) significantly improved these hematological parameters, alleviated central venous and sinusoidal congestion, increased T-SOD activity and upregulated *IL-10* mRNA expression. However, Vitamin U did not significantly reverse AFB_1_-induced elevation of ALP or reduction in GSH-Px, nor did it affect ALT, AST, or protein levels of the Nrf2/Keap1 pathway. Transcriptomic analysis revealed enrichment of DEGs in immune- and cell cycle-related pathways, with no direct enrichment observed in the Nrf2/Keap1 pathway. Vitamin U upregulated *Keap1* mRNA expression but did not alter KEAP1 protein levels. In conclusion, Vitamin U partially protects against AFB_1_-induced acute liver injury by ameliorating thrombocytopenia, vascular congestion, enhancing T-SOD activity, and upregulating *IL-10* expression, providing preliminary experimental evidence for further investigation.

## Simple Summary

Aflatoxin B_1_ (AFB_1_) is a prevalent feed contaminant in livestock production. It triggers oxidative stress and inflammation, ultimately leading to severe acute liver injury in animals. This study explored whether Vitamin U, a natural compound isolated from cruciferous vegetables, could alleviate AFB_1_-induced acute liver damage in mice. The results demonstrated that AFB_1_ exposure caused hepatic vascular congestion, reduced platelet and eosinophil counts, and inhibited the activity of the antioxidant enzyme glutathione peroxidase (GSH-Px). Vitamin U treatment partially restored these adverse changes: it restored platelet counts, relieved congestion in central veins and hepatic sinusoids, elevated total superoxide dismutase (T-SOD) activity, and upregulated the expression of the anti-inflammatory gene *IL-10*. Nevertheless, Vitamin U failed to significantly normalize the increased alkaline phosphatase (ALP) levels or recover the reduced GSH-Px activity, indicating that it only exerted partial hepatoprotective effects. Transcriptomic analysis revealed that Vitamin U mainly modulated immune-related pathways including the IL-17 and chemokine signaling pathways, as well as cell cycle pathways, rather than the classical Nrf2/Keap1 antioxidant pathway. In conclusion, Vitamin U confers partial protection against AFB_1_-induced acute liver injury via multiple mechanisms. This study provides evidence for exploring its molecular mechanisms and for future assessment of its potential against aflatoxin poisoning in livestock.

## 1. Introduction

Aflatoxin B_1_ (AFB_1_) is a potent hepatocarcinogen that contaminates grains and feed, posing a continuous threat to livestock health, welfare, and productivity [1]. The metabolism and detoxification of AFB_1_ occur primarily in the liver. In hepatocytes, AFB_1_ is activated by CYP1A2 and CYP3A4 to form the epoxide intermediate (AFBO), which extensively depletes reduced glutathione (GSH) and triggers leakage of the mitochondrial electron transport chain, leading to massive production of reactive oxygen species (ROS) and lipid peroxidation [2,3]. This oxidative stress further initiates endoplasmic reticulum stress and an inflammatory cascade, ultimately resulting in hepatocyte injury [4]. During this process, the nuclear factor erythroid 2-related factor 2 (Nrf2)-mediated antioxidant defense system is suppressed by GSH depletion, thereby weakening the organism’s intrinsic capacity to counteract AFB_1_ toxicity [5,6]. However, safe and efficient intervention approaches against AFB_1_-induced liver injury in vivo remain limited, highlighting the need to develop natural and effective detoxification agents.

Vitamin U (S-methylmethionine), as a sulfur-containing quaternary ammonium compound isolated from cruciferous plants, has long been classified as a “gastric ulcer healing factor”; recent pharmacological advances demonstrate it exerts multi-organ antioxidant and anti-inflammatory effects beyond gastric mucosal protection [7]. At the molecular level, Vitamin U can provide active methyl groups to participate in the methionine–threonine cycle, maintaining the level of S-adenosylmethionine to ensure the synthesis of glutathione; at the same time, it directly eliminates hydroxyl free radicals and superoxide anions, upregulates the expression of Nrf2 and its downstream glutathione peroxidase, superoxide dismutase, and catalase, and significantly inhibits the accumulation of malondialdehyde and protein carbonyls [7,8]. In terms of inflammation regulation, Vitamin U can block the TLR4/ NF-κB pathway, reduce the release of tumor TNF-α, IL-1β, and IL-6, thereby alleviating inflammatory infiltration and fibrotic remodeling in various experimental liver, kidney, and lung injuries [9,10]. Our previous research demonstrated that Vitamin U intervention alleviated AFB_1_-induced oxidative liver damage in a lactating mouse model [11]. However, that study focused on the special physiological stage of pregnancy and lactation. Whether Vitamin U exerts an antioxidant protective effect in ordinary adult mice following acute AFB_1_ gavage remains unknown.

Therefore, this study investigated the protective effect of Vitamin U against AFB_1_-induced acute liver injury in adult mice. The dose of Vitamin U (50 mg/kg), selected based on preliminary experiments, falls within the range reported to be hepatoprotective in rodent models of chemical-induced liver injury [12]. We assessed hematological parameters, serum biochemistry, hepatic histopathology, and oxidative stress markers, and used transcriptomic and molecular analyses to explore the underlying mechanisms, focusing on the Nrf2/Keap1 pathway and inflammatory regulation. This study provides preliminary experimental evidence to support the further evaluation of Vitamin U as a potential protective agent against AFB_1_-induced hepatotoxicity in mammalian systems.

## 2. Materials and Methods

### 2.1. Mouse Rearing and Experimental Design

All animal experimental procedures were conducted with the approval of the Animal Ethics Review Committee at Henan University of Animal Husbandry and Economy (approval No. HNUAHE ER 2324101, approval date: 15 March, 2024). Thirty 12-week-old ICR mice (41–45 g) with equal numbers of males and females were randomly assigned to five groups and housed under standard environmental conditions (24–26 °C, 12 h light/dark cycle) with free access to feed and water. Sex-related differences were not analyzed due to the acute experimental design and limited sample size. Prior to gavage, all mice underwent a 12 h fast with water available ad libitum. All treatments were delivered via a single intragastric administration. AFB_1_ was solubilized in 99.9% DMSO to formulate oral gavage stock solutions, whereas Vitamin U was dissolved in distilled water. Details of dosing regimens for each experimental group are described as follows: the AF group was intragastrically administered 3 mg/kg AFB_1_ prepared in 0.1 mL DMSO [12]; the U group received an oral dose of 50 mg/kg Vitamin U dissolved in 0.1 mL distilled water [8,10]; for the AU co-treatment group, equal aliquots of the AFB_1_-DMSO stock solution and aqueous Vitamin U solution were mixed to obtain a mixed dosing solution containing AFB_1_ at 3 mg/kg and Vitamin U at 50 mg/kg; the NC group received 0.1 mL undiluted 99.9% DMSO only, which corresponded to a DMSO exposure dose of approximately 2.44 g/kg and matched the solvent volume applied to the AF cohort; the MOCK group was given an equivalent 0.1 mL volume of distilled water. After 24 h, mice were anesthetized with 0.3% pentobarbital sodium (intraperitoneal) and euthanized by cervical dislocation for sample collection.

### 2.2. Sample Collection

At 24 h post-gavage, blood samples were collected via the orbital venous plexus. Serum was separated by centrifugation and stored for biochemical analysis. Liver tissues were harvested: a portion of the liver (consistent anatomical location across samples) was fixed for histological sectioning; the remaining liver tissue was snap-frozen in liquid nitrogen and stored at −80 °C for determination of oxidative stress indicators and transcriptome sequencing.

### 2.3. Determination of Blood Routine and Serum Biochemical Indicators

A fully automatic blood cell analyzer (BS-5000 VET, Shenzhen Mairui, Shenzhen, China) was used to analyze the whole blood samples from mice in each group. The specified indicators comprise indicators associated with white blood cells: total white blood cell count (WBC), neutrophil count (Neu) and percentage (Neu%), lymphocyte count (Lym) and percentage (Lym%), monocyte count (Mon) and percentage (Mon%), eosinophil count (Eos), and percentage (Eos%); indicators associated with red blood cells include red blood cell count (RBC), hemoglobin concentration (HGB), hematocrit (HCT), mean corpuscular volume (MCV), mean corpuscular hemoglobin (MCH), and mean corpuscular hemoglobin concentration (MCHC); indicators associated with platelets include platelet count (PLT); mean platelet volume (MPV); platelet distribution width (PDW); platelet crit percentage (PCT); red blood cell distribution width and coefficient of variation percentage (RDW-CV%), and standard deviation femtoliters (RDW-SD). 

Serum biochemical indicators related to liver function, including alanine transaminase (ALT), aspartate transaminase (AST), and alkaline phosphatase (ALP), were detected using an automatic biochemical analyzer (Model: BS-240VET, Shenzhen Mairui, Shenzhen, China).

### 2.4. Liver Tissue Sectioning

Liver tissues were fixed in formaldehyde for 12–24 h, rinsed with tap water, dehydrated through a graded ethanol series, cleared in xylene, embedded in paraffin, sectioned, and stained with hematoxylin and eosin (HE). Histopathological changes were examined in tissue sections under an optical microscope magnified at 400 times. For histopathological scoring, four non-consecutive liver sections per animal were examined, and ten randomly selected microscopic fields (400× magnification) were evaluated per section. Scoring was performed by two independent pathologists who were blind to the treatment groups. The two pathologists reviewed all slides independently and then reached a consensus for each parameter. The pathological scoring system was based on the previous literature [13]. Quantitative assessment was conducted based on the percentage area of various pathological changes: 0–10% was scored as 0 points, 10–25% as 1 point, 25–50% as 2 points, 50–75% as 3 points, and 75–100% as 4 points.

### 2.5. Measurement of Oxidative Stress Indicators

Oxidative stress indicators in liver tissues were measured using commercial kits (Beyotime Institute of Biotechnology, Shanghai, China), including malondialdehyde (MDA, cat. no. S0131) content, total superoxide dismutase (T-SOD, cat. no. S0101) activity, and glutathione peroxidase (GSH-Px, cat. no. S0057) activity.

### 2.6. Transcriptome Sequencing

Liver tissue samples were ground in liquid nitrogen, and total RNA was extracted using TRIzol reagent (Thermo Fisher, Waltham, MA, USA). RNA purity was detected using a Nanodrop 1C spectrophotometer (Thermo Fisher Scientific, Carlsbad, CA, USA) to ensure it met the experimental requirements, with the OD260/OD280 ratio ranging from 1.8 to 2.1 and the OD260/OD230 ratio ≥ 2.0. RNA integrity was assessed using an Agilent 2100 Bioanalyzer (Agilent Technologies, Santa Clara, CA, USA), and only samples with an RNA Integrity Number (RIN) ≥ 7.0 were used for subsequent sequencing. Strand-specific sequencing libraries were prepared using the VAHTS Universal V8 RNA-seq Library Prep Kit (Vazyme, Nanjing, China). Each treatment group contained six independent biological replicates, with 1 μg of the aforementioned high-quality total RNA used as the input sample. Paired-end 150 bp sequencing was performed on the Illumina NovaSeq 6000 (illumina, San Diego, CA, USA) platform. Raw sequencing data were subjected to base calling and quality filtering to produce high-quality reads. Processed data were aligned to the mouse reference genome (GRCm39) using Hisat2 (version v2.2.1). Gene expression levels were quantified using Feature Counts (version v2.0.3), and differentially expressed genes (DEGs) were identified using DESeq2 (version v1.44.0). Furthermore, KEGG pathway enrichment and GO functional annotation analyses of DEGs were performed using the DAVID Knowledgebase (version v2025_1). Protein–protein interaction (PPI) networks were constructed using the STRING database (version 12.5) and Cytoscape software (version 3.9.1).

### 2.7. Quantitative Real-Time PCR (RT-qPCR)

Total RNA from liver tissues (same batch as used for transcriptome sequencing) was reverse-transcribed into cDNA using the Thermo Fisher First-Strand cDNA Synthesis Kit. RT-qPCR was performed to detect the expression levels of DEGs and cytokine IL-6, TNF-α, and IL-10 in different groups using cDNA as templates. For detailed information on specific primer sequences, please refer to Supplementary Table S1. The 2^−ΔΔCt^ relative quantification method using GAPDH as the internal reference gene was used to analyze the target gene expression levels.

### 2.8. Western Blotting Analysis

Liver tissue protein extraction, quantification, SDS-PAGE, and Western blotting (WB) methods were performed as in our previous study [11]. Briefly, hepatic proteins were extracted with lysis buffer containing 1 mM PMSF (Beyotime Biotechnology, Shanghai, China). Protein concentrations were determined by the BCA assay, and equal protein amounts were separated on 4–20% gradient SDS-PAGE gels. Transferred membranes were incubated with primary antibodies targeting Nrf2 (Bioss, Beijing, China), Keap1, HMOX1, and β-actin (Proteintech, Rosemont, IL, USA) at 1:500 or 1:1000 dilutions, followed by IRDye-conjugated secondary antibodies. Full antibody information is listed in Supplementary Table S2. Protein bands were visualized using the Odyssey^®^ DLx system with Image Studio software (version 6.2; LI-COR Biosciences, Lincoln, NE, USA) and quantified via ImageJ 2, with β-actin as the internal control. All raw images were converted to 8-bit grayscale. Background correction was conducted via the built-in rolling ball algorithm (fixed radius = 50 pixels, light background mode). Uniform rectangular frames were applied to all target bands across all replicates. Quantitative metrics included area, mean gray value, min/max gray value, and integrated density; target protein signals were normalized to β-actin as the loading control. All blots were acquired with consistent exposure. Band identities were confirmed against pre-stained protein ladders based on theoretical molecular weights: NRF2 (68 kDa), KEAP1 (70 kDa), HMOX1 (30 kDa), β-actin (42 kDa). All image processing and quantification parameters remained unchanged across samples to reduce analytical error.

### 2.9. Data Processing

All statistical analyses were performed using SPSS 27.0. The Shapiro–Wilk test was used to verify data normality, and the Levene test was used to evaluate homogeneity of variance before subsequent statistical analysis. An independent samples *t*-test was used to compare two groups. For comparisons among multiple groups, one-way ANOVA coupled with Tukey’s post hoc test was used for data with normal distribution and homogeneous variance. For data that conformed to normal distribution but exhibited heterogeneous variance, Welch’s ANOVA was performed, followed by Tamhane’s T2 post hoc test. For data that did not follow a normal distribution but had consistent variances, the Kruskal–Wallis test with Bonferroni correction was used.

### 2.10. Sample Allocation and Exclusion Criteria

Sample allocation and exclusion criteria were as follows: Due to limited blood volume, hematology was performed on all animals (n = 6), while serum biochemistry (ALT, AST, ALP) was performed on four randomly selected mice per group with sufficient serum volume. For liver tissues, after fixation of the left lobe for histology, RNA-seq was prioritized and performed on all six mice per group (n = 6). Oxidative stress assays and RT-qPCR were performed on four randomly selected mice per group (n = 4) because the remaining tissue was insufficient for all animals. Western blotting was performed on the three mice with the greatest remaining tissue mass per group (n = 3). No samples were excluded due to outlier values or technical failure; the only reason for reduced sample sizes was insufficient sample volume for a given assay.

## 3. Results

### 3.1. Effects of Vitamin U on Blood Routine in Mice Induced by AFB_1_

For white blood cell-related indicators, the AF group had lower absolute counts of WBC, Neu, Lym, and Mon than the control groups, whereas the AU group showed values close to those of the MOCK and NC groups. Statistically, only the eosinophil count differed significantly: the AF group had a lower eosinophil count than the MOCK group, with no other significant differences among groups. Regarding red blood cell parameters, no significant differences were found in RBC, HGB, HCT, MCV, MCH, or RDW across groups. However, MCHC was significantly higher in the AU group than in the NC group, while other pairwise comparisons were not significant. For platelet-related indicators, both PLT and PCT showed significant differences. The AF group had lower PLT and PCT than the AU group. Other platelet parameters such as MPV and PDW did not differ significantly among groups.

In summary, AFB_1_ exposure was associated with marked reductions in Eos, PLT, and PCT, whereas Vitamin U intervention in the AU group was associated with significant improvements in these parameters, bringing PLT and PCT to levels comparable to or even higher than those in controls. These results suggest that Vitamin U alleviates AFB_1_-induced hematological toxicity (Table 1).

### 3.2. Effects of Vitamin U on Serum Transaminase Activities in Mice Induced by AFB_1_

**Figure 1:**
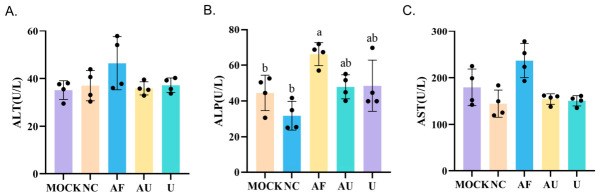
Effects of Vitamin U on serum transaminase activities in mice induced by AFB_1_. (**A**) Alanine transaminase (ALT) activity; (**B**) alkaline phosphatase (ALP) activity; (**C**) aspartate transaminase (AST) activity. Data are presented as mean ± SEM (n = 4). Different letters indicate significant differences (*p* < 0.05), and the same letters indicate no significant differences (*p* > 0.05).

To investigate the effect of Vitamin U on serum biochemical markers of liver function in mice with AFB_1_-induced liver injury, serum ALT, ALP, and AST activities were measured. The results showed no statistically significant differences in serum ALT activity among the groups (Figure 1A). Compared with the MOCK and NC groups, the AF group exhibited a significant increase in ALP activity. After Vitamin U intervention, the ALP levels in the AU group decreased compared with the AF group, but the difference between the groups was not statistically significant, and the ALP level in the AU group was also not significantly different from that in the control groups (Figure 1B). Regarding AST activity, the AF group showed higher values than the other groups, but no statistically significant differences were found among all groups (Figure 1C). In addition, there were no significant differences in ALT, ALP, or AST between the U group and the MOCK group. In summary, AFB_1_ treatment resulted in a significant elevation of serum ALP in mice, and Vitamin U supplementation showed a tendency to reduce ALP levels, although the effect did not reach statistical significance. Meanwhile, AFB_1_ had no significant effect on ALT or AST, and Vitamin U did not significantly modulate the changes in these two markers.

### 3.3. Effects of Vitamin U on Liver Tissue Morphology in Mice Induced by AFB_1_

**Figure 2:**
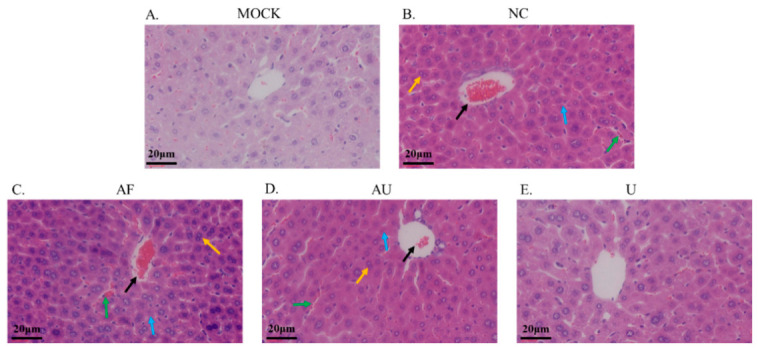
Pathological sections of the liver in mice induced by AFB_1_ and affected by Vitamin U (scale bar: 20 μm). (**A**) MOCK group (distilled water); (**B**) NC group (DMSO); (**C**) AF group (3 mg AFB_1_/kg body weight); (**D**) AU group (3 mg AFB_1_/kg body weight + 50 mg Vitamin U/kg body weight); (**E**) U group (50 mg Vitamin U/kg body weight). The black arrows denote congestion in the central vein, the green arrow highlights vasodilation and hyperemia within the sinusoids, the blue arrows signify hepatocellular degenerative alterations, and the orange arrows represent the occurrence of binucleated hepatocytes.

The H&E staining results revealed morphological changes in liver tissues of the different experimental groups. In both the MOCK and U groups, the liver tissue exhibited a complete structure, characterized by orderly arranged hepatic cords, clearly delineated liver sinusoids, and an absence of vascular dilation (Figure 2A,E). Compared with the MOCK group, NC group exhibited increased scores of hepatic vascular congestion, hepatocyte degeneration, and binucleated hepatocytes, with significant differences observed between the two groups (Figure 2B and Table 2). AFB_1_ exposure aggravated hepatic pathological damage in the AF group, which showed severe central venous hyperemia and sinusoidal dilatation and hyperemia, more serious hepatocyte degeneration, as well as excessive binucleated hepatocytes. All four pathological scores in the AF group were significantly higher than those in other groups. After Vitamin U intervention, hepatic congestion was obviously relieved in the AU group. Specifically, scores of central venous hyperemia and sinusoidal dilatation and hyperemia in the AU group were significantly lower than those in the AF group, while no significant differences were found in hepatocyte degeneration and binucleated hepatocyte formation between the two groups. In addition, no significant differences in all pathological indicators were observed between the AU group and NC group (Figure 2C and Table 2).

These results indicate that Vitamin U could effectively alleviate AFB_1_-induced hepatic congestion and hepatocellular damage, exerting a certain protective effect against AFB_1_-mediated liver injury in mice.

**Table 2:** The protective effects of Vitamin U on liver tissue in mice exposed to AFB_1_.

Groups/ Parameters	Hyperemia in the Vena Centralis	Dilatation and Hyperemia in Sinusoids	Degenerative Changes in Hepatocytes	Binucleated Hepatocyte Formations
MOCK	0.00 ± 0.00 ^c^	0.00 ± 0.00 ^c^	0.00 ± 0.00 ^c^	0.00 ± 0.00 ^b^
NC	1.29 ± 0.12 ^b^	0.63 ± 0.09 ^b^	0.71 ± 0.13 ^ab^	0.49 ± 0.07 ^ab^
AF	2.52 ± 0.40 ^a^	1.30 ± 0.13 ^a^	1.47 ± 0.18 ^a^	1.02 ± 0.14 ^a^
AU	1.28 ± 0.12 ^b^	0.51 ± 0.16 ^b^	0.69 ± 0.03 ^ab^	0.51 ± 0.04 ^ab^
U	0.00 ± 0.00 ^c^	0.00 ± 0.00 ^c^	0.00 ± 0.00 ^c^	0.00 ± 0.00 ^b^

Note: Data are presented as mean ± SEM (n = 4). Different letters indicate significant differences (*p* < 0.05), and the same letters indicate no significant differences (*p* > 0.05).

### 3.4. Effects of Vitamin U on Hepatic Oxidative Stress Markers in Mice Induced by AFB_1_

**Figure 3:**
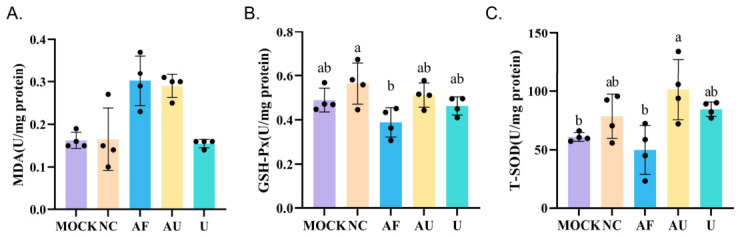
Effects of Vitamin U on hepatic oxidative stress markers in mice induced by AFB_1_. (**A**) Malondialdehyde (MDA) content; (**B**) total superoxide dismutase (T-SOD) activity; and (**C**) glutathione peroxidase (GSH-Px) activity. Data are presented as mean ± standard error (n = 4). Different letters indicate significant differences (*p* < 0.05), and the same letters indicate no significant differences (*p* > 0.05).

To evaluate the effects of Vitamin U on AFB_1_-induced hepatic oxidative stress, the levels of MDA, GSH-Px, and T-SOD were measured (Figure 3). As shown in Figure 3A, there were no statistically significant differences in MDA levels among the groups. Regarding GSH-Px activity, AFB_1_ treatment significantly decreased the enzyme activity. Although a slight increase was observed in the Vitamin U-treated group, the difference was not statistically significant compared with the AF group (Figure 3B). For T-SOD activity, the AF group showed only a slight decrease compared with the MOCK and NC groups, which did not reach statistical significance (Figure 3C). However, T-SOD activity was significantly increased in the Vitamin U-treated group compared with the AF group. Collectively, these results indicate that Vitamin U had no significant effect on the changes in MDA levels or the decrease in GSH-Px activity induced by AFB_1_, but significantly increased T-SOD activity.

### 3.5. Effects of Vitamin U on Transcriptome Profile in AFB_1_-Induced Mouse Liver Tissues

After quality filtering, all samples obtained sufficient clean reads. Each sample yielded clean reads ranging from 31.6 million to 119.1 million, and all samples exhibited high sequencing quality with clean Q30 values above 95%. The average total mapping rate against the mouse reference genome GRCm39 exceeded 97%, and the average unique mapping rate surpassed 85%. Hierarchical clustering of sample-to-sample distances confirmed high reproducibility within each treatment group, as illustrated in Figure S1.

**Figure 4:**
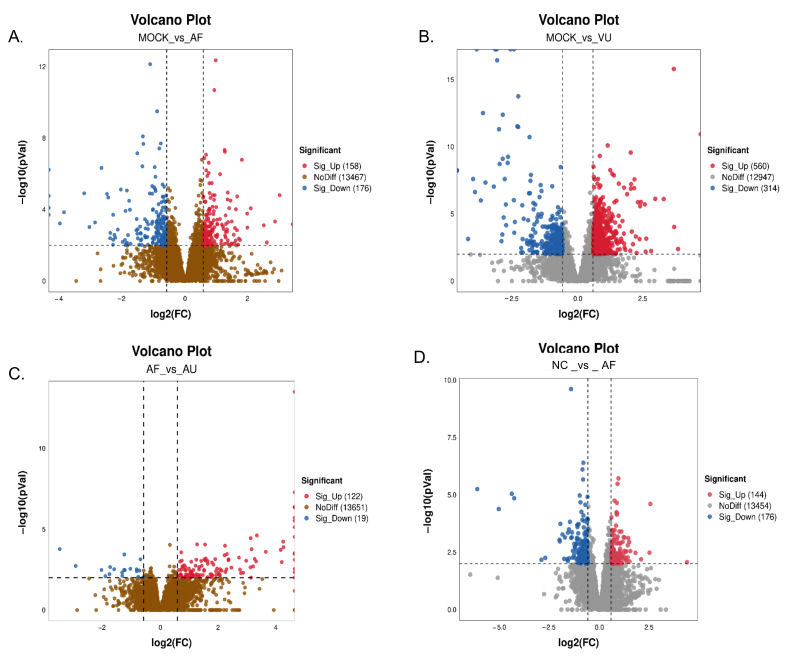
Volcano plots of hepatic DEGs. DEG screening thresholds FC > 1.5 and *p* < 0.01. (**A**) MOCK vs. AF group; (**B**) MOCK vs. VU group; (**C**) AF vs. AU group; (**D**) NC vs. AF group. Red dots: significantly upregulated genes; blue dots: significantly downregulated genes; brown or gray dots: non-differentially expressed genes.

Differentially expressed genes (DEGs) were analyzed using DESeq2 v1.44.0, with the screening criteria set as FC > 1.5 and *p* < 0.01. The results of differential expression analysis showed that compared with the MOCK group, the AF group had 334 DEGs, including 158 upregulated and 176 downregulated genes (Figure 4A). In the comparison between the MOCK and U groups, 874 DEGs were identified, including 560 upregulated and 314 downregulated genes (Figure 4B). When comparing the AF group with the AU group, 141 DEGs were identified, including 122 genes upregulated and 19 downregulated genes (Figure 4C). In the comparison between the NC and the AF groups, a total of 320 DEGs were identified, including 144 upregulated and 176 downregulated genes (Figure 4D). These results indicated that both the AF and AU treatment groups significantly affected the liver gene expression profiles in mice, and the AU group showed greater changes.

**Figure 5:**
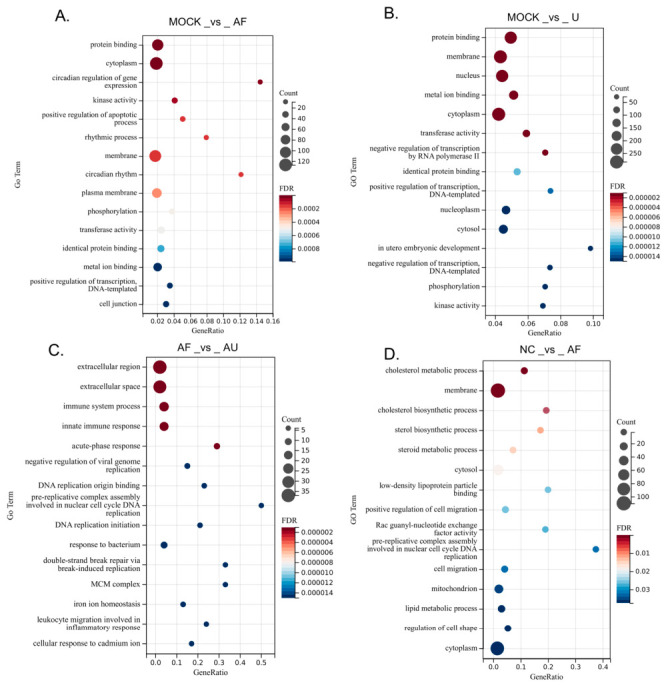
GO functional enrichment analysis of DEGs, with FDR < 0.05. (**A**) MOCK vs. AF group; (**B**) MOCK vs. VU group; (**C**) AF vs. AU group; (**D**) NC vs. AF group. The top 15 significantly enriched GO terms are presented for each comparison. Bubble size indicates the number of enriched genes, and bubble color represents the false discovery rate, with red indicating greater significance.

GO functional enrichment analysis of DEGs in each group was performed using DAVID. The results showed that the DEGs between the MOCK and AF groups were significantly enriched in rhythm regulation-related functions, including the regulation of cytoplasmic circadian rhythm gene expression and circadian rhythm (Figure 5A). DEGs between the MOCK and U groups were mainly involved in gene expression regulation processes, with significant enrichment in molecular functions, such as protein binding and metal ion binding, as well as biological processes such as negative regulation of RNA polymerase II transcription and protein phosphorylation (Figure 5B). DEGs between the AF and AU groups exhibited significant immune-related characteristics, including biological processes such as the immune system process and innate immune response, as well as specific immune functions such as acute-phase response and negative regulation of viral genome replication (Figure 5C). DEGs between the NC and AF groups were mainly associated with metabolism and signal transduction, including the chemokine signaling pathway and the steroid biosynthesis pathway (Figure 5D).

**Figure 6:**
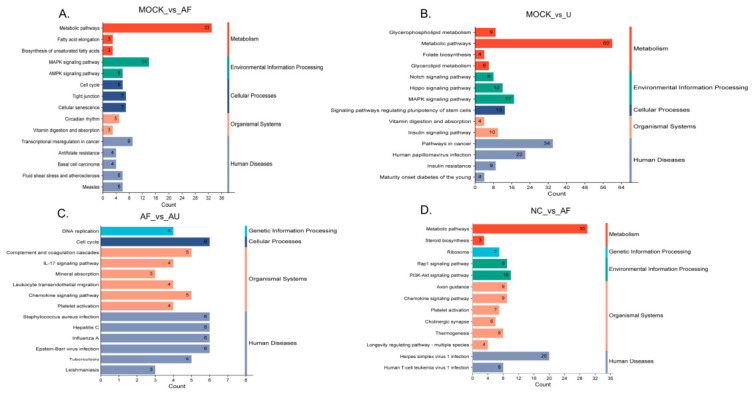
KEGG enrichment analysis of DEGs from different comparison groups (FDR < 0.05). (**A**) MOCK vs. AF group; (**B**) MOCK vs. VU group; (**C**) AF vs. AU group; (**D**) NC vs. AF group. Significantly enriched KEGG pathways are shown for each comparison, with gene counts indicated at the end of each bar.

KEGG functional enrichment analysis (FDR < 0.05) of DEGs in each group showed that DEGs between the MOCK group and the AF group were mainly enriched in lipid metabolism, such as fatty acid elongation, biosynthesis of unsaturated fatty acids, and circadian rhythm-related pathways (Figure 6A). DEGs between the MOCK and U groups were significantly associated with the Notch/Hippo pathway (which regulates stem cell pluripotency) and with metabolic processes, such as glycerophospholipid metabolism and folate biosynthesis (Figure 6B). DEGs between the AF and AU groups were enriched in cell proliferation, including DNA replication, and cell cycle and immune inflammatory responses, including the IL-17 signaling pathway, and the chemokine signaling pathway (Figure 6C). DEGs between the NC and AF groups were characterized by metabolic reprogramming, such as steroid synthesis, as well as by activation of ribosomes and viral infection pathways (Figure 6D). These results indicated that Vitamin U may antagonize AFB_1_-induced hepatotoxicity by regulating pathways related to metabolism, immunity, and the cell cycle, providing transcriptomic evidence for its hepatoprotective mechanism. Notably, although the canonical Nrf2/Hmox1 antioxidant pathway was not significantly enriched in the above GO and KEGG analyses, multiple DEGs are associated with oxidative stress response, redox balance, and inflammatory regulation, implying potential crosstalk between transcriptomic changes and the core antioxidant signaling pathway.

**Figure 7:**
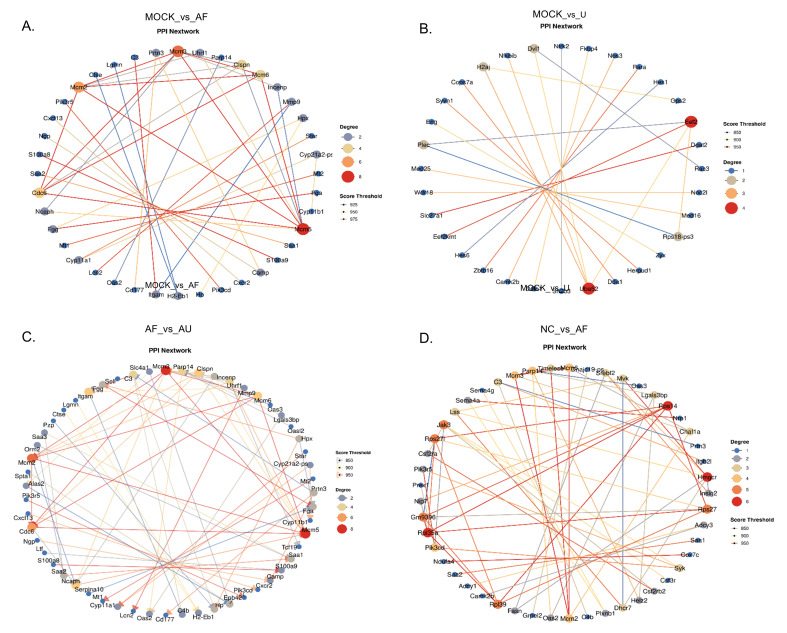
PPI network analyses of differential omics features across comparison groups. (**A**) MOCK vs. AF group; (**B**) MOCK vs. VU group; (**C**) AF vs. AU group; (**D**) NC vs. AF group. Protein–protein interaction network analysis, with confidence level > 0.4. Larger and darker nodes denote higher connectivity; thicker and darker edges indicate stronger protein interactions.

PPI network analysis using the STRING database identified core regulatory modules of DEGs in each group. The analysis showed that for the MOCK vs. AF comparison group, the core interaction module centered on genes *MCM2*, *MCM3*, *MCM5,* and *CDC6* (Figure 7A). The network for the MOCK vs. U group was constructed around genes *Eef2* and *UBA52* (Figure 7B). The network for the AF vs. AU group was constructed around genes such as *MCM2*, *MCM3*, *MCM5*, and *CDC6* (Figure 7C). The network for the NC vs. AF group was constructed around genes *RPS14*, *HMGCR*, and *RPL35a* (Figure 7D).

**Figure 8:**
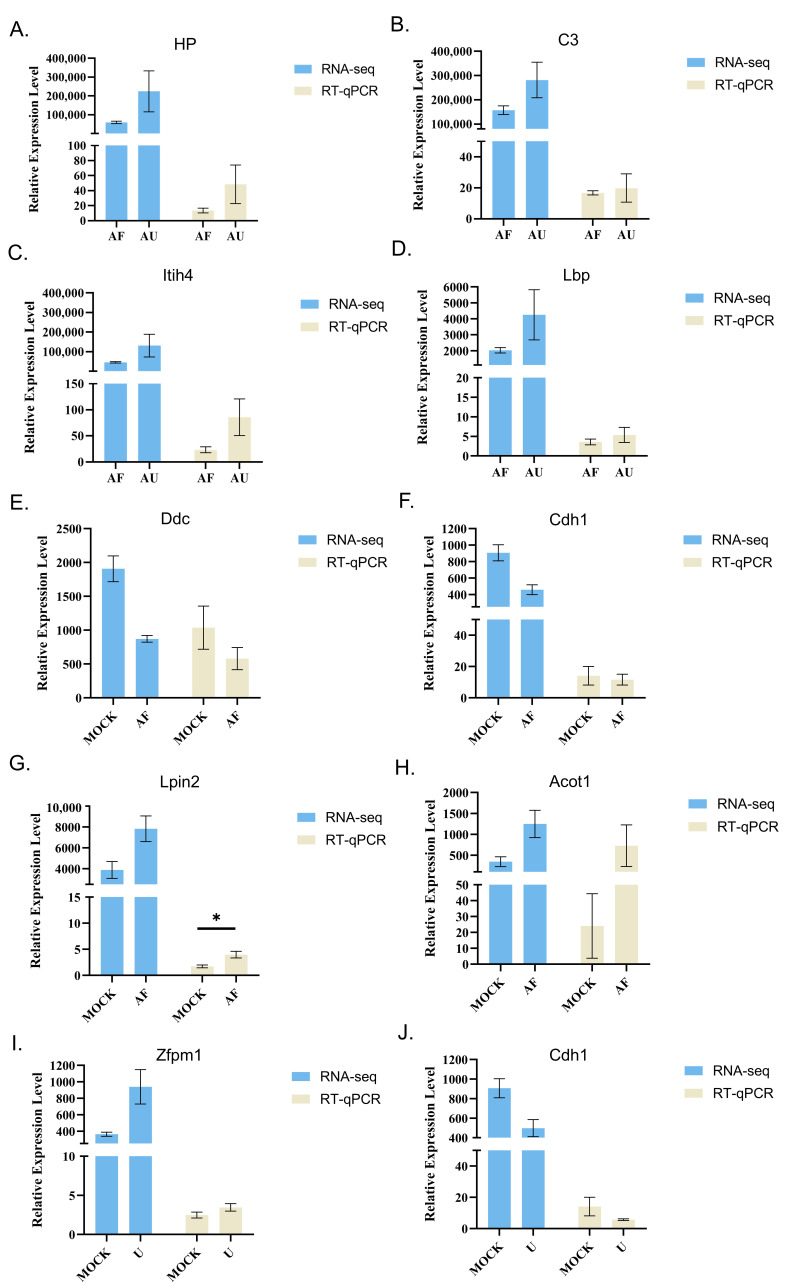
Verification of differentially expressed genes in different experimental groups using RT-qPCR. (**A**) HP gene; (**B**) C3 gene; (**C**) Itih4 gene; (**D**) Lbp gene; (**E**) Ddc gene; (**F**,**J**) Cdh1 gene; (**G**) Lpin2 gene; (**H**) Acot1 gene and (**I**) Zfpm1 gene. * indicates significant difference (*p* < 0.05).

Ten candidate genes were randomly selected from the DEGs of the AF vs. AU group, MOCK vs. AF group, and MOCK vs. U group, and quantitative real-time PCR (RT-qPCR) was used to verify the accuracy of the RNA-seq results. As shown in Figure 8, the RT-qPCR results were consistent with those of RNA-seq, indicating the accuracy of the sequencing results.

### 3.6. Effects of Vitamin U on the Expression of Nrf2 Signaling Pathway-Related Proteins in the Livers of Mice Induced by AFB_1_

**Figure 9:**
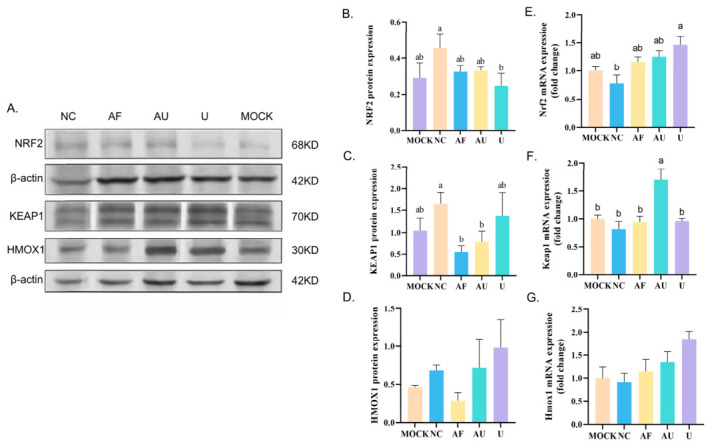
Effect of Vitamin U on protein and mRNA expression of genes related to the Nrf2 signaling pathway in AFB_1_-induced mouse liver. (**A**) The protein expression of NRF2, KEAP1, and HMOX1 in the liver was detected by Western blot; (**B**) NRF2, (**C**) KEAP1, and (**D**) HMOX1 protein bands were quantified utilizing the density method. (**E**) *Nrf2*, (**F**) *Keap1*, and (**G**) *Hmox1* mRNA expression levels were quantified utilizing RT-qPCR. Mice were treated as follows: MOCK (distilled water), NC (DMSO), AF (3 mg AFB_1_/kg b.w./day), AU (3 mg AFB_1_/kg b.w./day + 50 mg Vitamin U/kg b.w./day), U (50 mg Vitamin U/kg b.w./ day). Data are presented as mean ± SEM (n = 3, protein; n = 4, mRNA). Different letters indicate significant difference (*p* < 0.05), while the same letter indicates non-significant differences (*p* > 0.05).

Based on the well-established role of the Nrf2/Hmox1 pathway in antioxidant defense and hepatoprotection against mycotoxin-induced liver injury, combined with the aforementioned oxidative stress phenotypes, the protein and mRNA expression of key molecules in this pathway were examined to elucidate the regulatory mechanism of Vitamin U on AFB_1_-induced acute liver injury. The results showed that no statistically significant differences in NRF2 protein or mRNA expression levels were observed among the experimental groups, regardless of AFB_1_ or Vitamin U treatment (Figure 9B,E). For KEAP1, AFB_1_ treatment significantly decreased KEAP1 protein levels compared with the NC group (Figure 9C). The addition of Vitamin U did not significantly alleviate this reduction, and KEAP1 protein levels remained generally consistent with those in the AF group. Notably, at the mRNA level, Vitamin U treatment resulted in a significant upregulation of *Keap1* expression compared with both the AF and NC groups (Figure 9F). For *Hmox1*, no significant differences in protein or mRNA expression levels were detected among the groups (Figure 9D,G).

Collectively, these results indicated that AFB_1_ exposure significantly downregulated KEAP1 protein expression without affecting NRF2 or HMOX1 levels. Vitamin U treatment did not reverse the AFB_1_-induced decrease in KEAP1 protein but significantly upregulated Keap1 mRNA expression (the original western blot pictures can be found in File S1).

### 3.7. Analysis of Inflammatory Cytokine mRNA Expression Profiles

**Figure 10:**
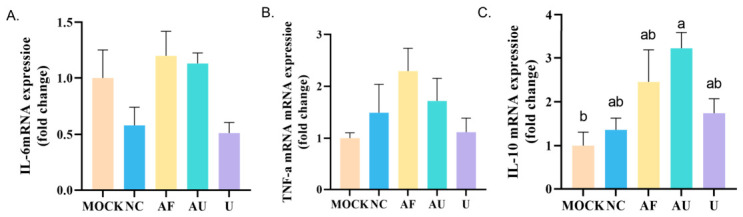
Cytokine mRNA expression profiles. (**A**) *IL-6*, (**B**) *TNF-α*, and (**C**) *IL-10* mRNA expression levels were quantified utilizing RT-qPCR. Data are presented as mean ± SEM (n = 4). Different letters indicate significant difference (*p* < 0.05), while the same letter indicates non-significant differences (*p* > 0.05).

This study detected the mRNA expression of three key inflammatory cytokines, namely *IL-6*, *TNF-α*, and *IL-10*, in different treatment groups to evaluate the inflammatory response in liver injury induced by AFB_1_ (Figure 10). As shown in the results, no significant intergroup differences in hepatic *IL-6* mRNA expression were observed. Although the *TNF-α* mRNA levels peaked in the AF group, pairwise comparisons revealed no statistically significant differences across the experimental groups. Compared with the NC group, *IL-10* mRNA abundance was numerically elevated in the AF group; this difference was not statistically significant. After Vitamin U supplementation, the *IL-10* transcriptional level was further upregulated in the AU group, which was markedly higher than those in the MOCK, NC, and U groups, while no significant difference existed between the AU and AF groups.

These data indicated that single AFB_1_ exposure did not significantly increase hepatic *IL-10* expression, whereas combined intervention with Vitamin U markedly increased *IL-10* transcription. In addition, neither AFB_1_ treatment nor Vitamin U supplementation exerted significant regulatory effects on *IL-6* and *TNF-α* mRNA expression.

## 4. Discussion

Aflatoxin B_1_ (AFB_1_) is a widespread hepatotoxic contaminant in feed and food, which severely impairs livestock health. The present study systematically evaluated the hepatoprotective efficacy of Vitamin U against AFB_1_-triggered acute hepatic injury in mice. Our results demonstrated that Vitamin U partially ameliorated AFB_1_-caused hematological abnormalities, alleviated histopathological hepatic lesions, elevated hepatic T-SOD activity, and upregulated hepatic mRNA expression of the anti-inflammatory cytokine IL-10. Transcriptomic profiling further indicated that Vitamin U likely exerts hepatoprotective effects by modulating signaling pathways involved in lipid metabolism, immune-inflammatory responses, and cell cycle progression. However, Vitamin U did not significantly alter serum ALT, AST, hepatic MDA, and GSH-Px levels, and no obvious changes were observed in the protein expression of the Nrf2/Hmox1 pathway.

Accumulating evidence indicates that AFB_1_ exposure impairs host immune function, particularly cell-mediated immune responses, and is accompanied by reduced counts of total leukocytes, lymphocytes, and monocytes [14,15]. In this study, AFB_1_ administration markedly reduced the absolute eosinophil count, accompanied by prominent declines in PLT and PCT. The decreased eosinophil abundance may be attributed to AFB_1_-mediated suppression of bone marrow hematopoiesis or accelerated eosinophilic apoptosis. Reduced platelet numbers presumably stem from suppressed megakaryocytic function in bone marrow and excessive platelet consumption triggered by vascular endothelial damage upon AFB_1_ intoxication [16]. Notably, PLT and PCT levels were significantly higher in the AU group relative to the AFB_1_-treated AF group. After Vitamin U treatment, PLT and PCT recovered to levels comparable to or even higher than those in the blank group, confirming its protective effect against AFB_1_-induced thrombocytopenia. These observations align with earlier reports on the bone marrow-protective property of Vitamin U [7], and its molecular mechanisms need further research.

Serum ALT, AST, and ALP serve as canonical biochemical markers for hepatic function assessment [17]. In the current study, AFB_1_ exposure induced a significant elevation in serum ALP activity, despite non-significant increases in ALT and AST concentrations. This phenomenon partially conflicts with existing published data. Multiple in vivo trials reported markedly elevated serum ALT, AST, and ALP levels following acute AFB_1_ intoxication in mice [18,19], whereas other investigations demonstrated dose and time-dependent hepatotoxic responses to AFB_1_, and only ALP changed significantly under our experimental conditions [20]. The distinct ALP upregulation suggests that AFB_1_ preferentially damages biliary epithelial cells, triggering cholestatic hepatic impairment [21]. Although ALP concentrations tended to drop in the AU group compared with the AF group following Vitamin U treatment, no intergroup statistical difference was attained, and ALP levels remained comparable to the blank control, implying limited potency of Vitamin U in reversing elevated ALP. Non-significant differences in ALT and AST across all experimental groups may be explained by the adopted AFB_1_ dosage (3 mg/kg) and single sampling time point (24 h post-administration), which failed to induce substantial necrotic damage of hepatocytes. In addition, mild hepatic pathological lesions were detected in the DMSO vehicle NC group, consistent with previous findings that high-dose DMSO elicits mild hepatotoxicity [22]. Since an identical DMSO dosage was applied to both AF and AU groups, DMSO-induced mild liver injury exerted negligible confounding effects on intergroup statistical comparisons.

Histopathological liver examination is regarded as the gold standard for evaluating AFB_1_ toxicity and candidate hepatoprotective agents [18,19]. In our study, AFB_1_ intoxication resulted in severe central venous congestion, sinusoidal dilation and congestion, hepatocellular degeneration, and increased binucleated hepatocytes, accompanied by drastically elevated pathological scoring relative to the blank control group. After Vitamin U intervention, significantly reduced scores for central venous congestion and hepatic sinusoidal congestion were observed in the AU group compared with the AF group, confirming that Vitamin U effectively relieves AFB_1_-caused hepatic hemodynamic disorders and microcirculatory dysfunction. By contrast, no statistical differences in hepatocellular degeneration and binucleation scores existed between AU and AF groups, demonstrating that Vitamin U confers stronger protection against hepatic vascular lesions than parenchymal hepatocyte injury. Such divergent protective outcomes imply cell-type-selective hepatoprotection of Vitamin U within hepatic tissue. The liver structure remained normal in the blank and Vitamin U monotherapy groups, indicating that Vitamin itself has no hepatotoxicity.

Oxidative stress constitutes a core pathological mechanism underlying AFB_1_-mediated hepatotoxicity [1]. AFB_1_ is metabolized by hepatic CYP450 enzymes to form toxic products, which consume intracellular GSH, induce ROS accumulation, and exacerbate lipid peroxidation [2]. Three representative oxidative stress indicators including MDA, T-SOD, and GSH-Px were quantified herein. AFB_1_ exposure significantly suppressed hepatic GSH-Px activity, whereas hepatic MDA content and T-SOD activity remained statistically unchanged relative to the blank control group. Vitamin U supplementation markedly restored T-SOD activity in the AU group versus the AF group, with non-significant variations in MDA and GSH-Px levels. The above antioxidant tendency coincides with prior studies documenting Vitamin U-mediated antioxidant capacity across distinct hepatic injury models [8,11]. Remarkably enhanced T-SOD activity reveals that Vitamin U eliminates excess superoxide anion to mitigate hepatic oxidative insults. The non-significant changes in MDA and GSH-Px were likely related to AFB_1_ dosage, post-exposure sampling time, limited sample size (n = 4 per group), and insufficient detection sensitivity of adopted assays. Additionally, relatively large standard deviation for detected oxidative stress indices indicated prominent inter-individual biological variation, which potentially masked genuine therapeutic effects of Vitamin U.

Transcriptome sequencing results revealed profound remodeling of hepatic transcriptional profiling upon AFB_1_ insult, with DEGs predominantly enriched in pathways governing lipid metabolism and immune-inflammatory responses, consistent with established toxicological paradigms of AFB_1_ [23,24]. Specifically, DEGs identified from the AF vs. NC comparison were significantly enriched in fatty acid elongation, unsaturated fatty acid biosynthesis, and steroid biosynthesis pathways, consistent with the well-documented property of AFB_1_ to trigger lipid accumulation and systemic lipid metabolic perturbation in the liver [25,26]. By contrast, DEGs identified in the AF vs. AU comparison were enriched in biological processes related to immune system regulation, innate immune response, and acute-phase reaction, alongside IL-17 and chemokine-mediated signaling cascades, substantiating that Vitamin U antagonizes AFB_1_ hepatotoxicity via transcriptional reprogramming of immune-inflammatory related genes. Furthermore, DEGs from AF vs. AU contrast were enriched in DNA replication and cell cycle pathways, suggesting that Vitamin U may regulate hepatocyte cycle and proliferation. Subsequent PPI network analysis identified MCM2, MCM3, MCM5, and CDC6 (core DNA replication-associated genes) as central hub genes in both MOCK vs. AF and AF vs. AU interaction modules, further consolidating the regulatory role of Vitamin U on hepatocellular cycle progression.

The canonical Nrf2/Keap1 signaling cascade serves as a master defensive axis against intracellular oxidative stress [27,28]. In the present study, AFB_1_ exposure significantly downregulated KEAP1 protein abundance relative to the group, while the protein levels of NRF2 and HMOX1 remained unchanged. KEAP1 acts as a negative regulator of NRF2. A decrease in its expression should lead to an accumulation of NRF2 protein and increased in its nuclear translocation, thereby upregulating the expression of downstream antioxidant genes, including Hmox1 [29]. Unexpectedly, the anticipated regulatory cascade was not reproduced in the AFB_1_-challenged AF group; instead, NRF2 and HMOX1 protein levels showed marginal declines without statistical significance. This contradictory observation implies that AFB_1_ accelerates NRF2 ubiquitination and proteasomal degradation independent of KEAP1, possibly via alternative E3 ubiquitin ligases such as β-TrCP and Hrd1 [30,31].

At the transcriptional level, Vitamin U supplementation significantly elevated Keap1 mRNA abundance in the AU group compared with AF and NC groups, accompanied by an increasing trend in Nrf2 mRNA expression. Such transcriptional alternations comply with classic ARE-triggered negative feedback loop: activated nuclear NRF2 binds ARE motifs within promoter regions of Nrf2 and Keap1 genes to initiate their own transcription for feedback restriction of pathway overactivation [5,32]. Nevertheless, elevated Keap1 mRNA failed to translate into increased KEAP1 protein in the AU group, and HMOX1 protein only showed non-significant mild upregulation after Vitamin U intervention. Three plausible interpretations are proposed for this transcription–translation decoupling phenomenon: first, the single 24 h post-exposure sampling time point might miss transient peak protein expression following transcriptional activation; second, AFB_1_-mediated post-translational modifications or accelerated protein turnover counteract transcriptional upregulation of target genes; third, total cellular protein extracted for Western blot cannot mirror Nrf2 functional status, as Nrf2 biological activity largely relies on its nuclear translocation efficiency rather than total cytoplasmic protein content [6]. Subsequent experiments can combine nucleocytoplasmic fractionation and immunofluorescence staining to directly quantify the nuclear translocation of Nrf2. Our previous in vivo data from a gestation–lactation mouse model documented prominent Vitamin U-triggered upregulation of hepatic Nrf2 and Hmox1 protein [11], which conflicts with current acute intoxication outcomes. Such inconsistency is likely derived from divergent physiological status of experimental animals (gestating/lactating versus adult mice), distinct AFB_1_ exposure regimens (subchronic lactational transmission versus single acute intragastric administration), and varied Vitamin U dosage and administration duration, indicating that host physiological conditions and toxicant exposure paradigms profoundly determine the final hepatoprotective performance of Vitamin U.

Hepatic inflammatory infiltration represents a pivotal pathogenic driver of AFB_1_-induced liver damage [33]. Our qPCR data demonstrated markedly boosted *IL-10* mRNA expression in the AU group versus MOCK, NC, and U groups following Vitamin U intervention. In contrast, mRNA levels of pro-inflammatory mediators *IL-6* and *TNF-α* remained unchanged across all experimental cohorts. As a canonical anti-inflammatory cytokine, IL-10 restrains the excessive inflammatory cascade via suppressing macrophage activation and downstream pro-inflammatory cytokine secretion to alleviate progressive tissue injury [4]. Significant transcriptional induction of *IL-10* therefore reveals that Vitamin U alleviates AFB_1_ hepatotoxicity predominantly by reinforcing endogenous anti-inflammatory signaling. However, the lack of obvious *IL-6* and *TNF-α* transcriptional elevation after AFB_1_ exposure may result from insufficient activation of canonical NF-κB cascade under the current AFB_1_ dosage and 24 h single-time sampling scheme. Consistently, KEGG enrichment based on AF/AU DEGs highlighted altered IL-17 and chemokine signaling pathways, which dominate neutrophil recruitment and Th17 cell differentiation during inflammatory progression []. Collectively, Vitamin U modulates hepatic inflammatory responses preferentially by enhancing anti-inflammatory *IL-10* signaling instead of suppressing transcription of canonical pro-inflammatory cytokines *IL-6* and *TNF-α.* These findings are partially consistent with previously reported anti-inflammatory potency of Vitamin U in extrahepatic organ injury models including renal and pulmonary toxic damage [9,10], while downstream molecular cascades underlying Vitamin U-mediated inflammatory regulation remain to be validated mechanistically.

Notably, phytogenic compounds have emerged as promising countermeasures against AFB_1_ toxicity. A previous study demonstrated that curcuminoids in dairy cows altered AFB_1_ metabolite excretion of AFM1 and aflatoxicol, implicating modulation of AFB_1_ metabolism and oxidative stress [34]. Combined with the present mouse-derived results, this work only provides a basic theoretical reference for the research of plant-derived anti-aflatoxin substances; long-term toxicity and efficacy experiments on target livestock need to be carried out separately to judge its practical feeding application prospect.

### Limitations

This study has several limitations. Limited liver tissue resulted in inconsistent sample sizes across assays (n = 4 for serum biochemistry and oxidative stress assays, n = 3 for Western blot), which may lower statistical power and account for the non-significant trends of GSH-Px and ALP. We only measured MDA, T-SOD, and GSH-Px, and did not detect ROS, CAT, and total GSH, hindering a comprehensive analysis of the antioxidant mechanism.

This work adopted an acute single-dose model with sampling conducted at 24 h post-treatment. Only one dose of AFB_1_ and Vitamin U was used, so time-dependent responses and dose–effect relationships were not investigated. We also combined male and female mice without analyzing sex differences in hepatic responses.

Evidence for the Nrf2/Hmox1 pathway remains incomplete. We only measured total protein levels rather than Nrf2 nuclear translocation. Inflammatory responses were assessed at the mRNA level alone, without protein validation via ELISA. AFB_1_ metabolites and DNA adducts, key biomarkers for genotoxicity, were also not examined, leading to insufficient evidence for the pathway regulation. The Vitamin U dosage adopted herein was determined based on preliminary pre-experiments and published studies [11,35], whereas optimal therapeutic equivalent dosage and in vivo safety profiles for target livestock species require systematic dose–effect trials for translational application.

## 5. Conclusions

In summary, Vitamin U partially mitigates acute AFB_1_-induced hepatotoxicity in mice by ameliorating PLT, PCT, and EOS, reducing hepatic vascular injury, boosting hepatic T-SOD activity, and upregulating anti-inflammatory *IL-10* transcription. Transcriptomics reveals Vitamin U antagonizes AFB_1_ toxicity via regulating lipid metabolism, inflammation, and cell cycle pathways. Despite unchanged Nrf2/Keap1 protein levels, Vitamin U upregulates *Keap1* at the mRNA level, suggesting transcriptional regulation of this antioxidant pathway. This study preliminarily verified the partial hepatoprotective effects of Vitamin U against acute AFB_1_-induced liver injury. Nevertheless, systematic long-term feeding trials on target livestock species are indispensable before any practical breeding application can be proposed.

## Figures and Tables

**Table 1:** Routine blood test results of mice in different treatment groups.

Indicator	MOCK	NC	AF	AU	U
WBC (10^9^/L)	4.20 ± 0.43	4.06 ± 0.58	2.50 ± 0.56	4.39 ± 0.52	4.16 ± 0.76
Neu (10^9^/L)	0.89 ± 0.13	0.91 ± 0.27	0.44 ± 0.14	1.31 ± 0.48	0.85 ± 0.40
Lym (10^9^/L)	2.77 ± 0.37	2.79 ± 0.35	1.84 ± 0.39	2.59 ± 0.41	2.97 ± 0.49
Mon (10^9^/L)	0.27 ± 0.05	0.21 ± 0.06	0.13 ± 0.05	0.33 ± 0.15	0.21 ± 0.04
Eos (10^9^/L)	0.27 ± 0.05 ^a^	0.15 ± 0.02 ^ab^	0.09 ± 0.01 ^b^	0.15 ± 0.02 ^ab^	0.12 ± 0.01 ^ab^
Neu (%)	21.57 ± 3.27	20.7 ± 4.47	16.92 ± 2.37	27.92 ± 7.19	18.97 ± 5.33
Lym (%)	65.47 ± 3.77	70.53 ± 5.10	73.62 ± 3.74	61.43 ± 8.83	72.12 ± 6.31
Mon (%)	6.43 ± 0.60	4.85 ± 0.99	5.13 ± 1.09	6.87 ± 2.16	5.45 ± 0.98
Eos (%)	6.53 ± 1.17	3.92 ± 0.47	4.33 ± 0.70	3.78 ± 0.62	3.47 ± 0.65
RBC (10^12^/L)	8.70 ± 0.35	8.83 ± 0.69	9.56 ± 0.32	7.91 ± 0.90	9.38 ± 0.31
HGB (g/L)	143.17 ± 4.69	146.33 ± 12.16	157.00 ± 4.49	133.67 ± 13.11	151.00 ± 4.73
HCT (%)	39.08 ± 1.43	40.05 ± 3.05	42.07 ± 1.41	29.38 ± 6.03	40.97 ± 1.09
MCV (fL)	45.00 ± 0.56	45.42 ± 0.20	44.05 ± 0.51	45.17 ± 1.13	43.75 ± 0.64
MCH (pg)	16.52 ± 0.18	16.57 ± 0.15	16.45 ± 0.21	17.17 ± 0.56	16.12 ± 0.23
MCHC (g/L)	366.50 ± 3.08 ^ab^	364.50 ± 3.15 ^b^	373.83 ± 3.3 ^ab^	379.83 ± 5.01 ^a^	368.67 ± 3.13 ^ab^
RDW-CV (%)	16.85 ± 0.45	16.97 ± 0.49	16.10 ± 0.66	18.12 ± 1.29	16.63 ± 0.55
RDW-SD (fL)	34.43 ± 1.29	35.07 ± 1.06	32.22 ± 1.17	37.18 ± 2.55	33.23 ± 0.90
PLT (10^9^/L)	718.5 ± 86.99 ^ab^	763.17 ± 151.22 ^ab^	401.67 ± 79.68 ^b^	913.67 ± 161.56 ^a^	696.17 ± 104.73 ^ab^
MPV (FL)	4.71 ± 0.15	4.97 ± 0.22	5.02 ± 0.11	5.20 ± 0.19	4.77 ± 0.09
PDW	15.70 ± 0.11	15.58 ± 0.09	15.68 ± 0.11	15.78 ± 0.13	15.41 ± 0.04
PCT (%)	0.34 ± 0.05 ^ab^	0.39 ± 0.09 ^ab^	0.20 ± 0.04 ^b^	0.49 ± 0.10 ^a^	0.33 ± 0.05 ^ab^

Note: Data are presented as mean ± SEM (n = 6). Different letters indicate significant differences (*p* < 0.05), and the same letters indicate no significant differences (*p* > 0.05).

## Data Availability

The data presented in this study are openly available in the SRA database, and the BioProject accession number is PRJNA1308316.

## Supplementary Materials

The following are available online at https://www.mdpi.com/article/10.3390/vetsci13070621/s1, Table S1: Primer sequences of qRT-PCR, Table S2: List of antibodies used for western blot analysis, File S1: Original images of Western-blotting.
