# Supplementary material for: Vitamin U Attenuates Acute Aflatoxin B1-Induced Liver Injury in Mice: Biochemical, Histological and Transcriptomic Evidence
Source: Vet Sci. 2026 Jun 26;13(7):621. doi: 10.3390/vetsci13070621 (PMC13431570; doi:10.3390/vetsci13070621)
Supplement: Supplementary file 1 [file vetsci-13-00621-s001.zip › vetsci-4317631-supplementary tables.pdf]

Supplementary Table S1 Primer sequences of qRT-PCR.

Primer	Sequence (5'-3')
mmu-Hp-F	ATCGCTGCCGACAGTTCTAC
mmu-Hp-R	CTCTCCAGCGACTGTGTTCA
mmu-Itih4-F	GATCATCGATGGCGTGACCT
mmu-Itih4-R	AGTGGTCTTGACGATGCCAG
mmu-Lbp-F	GGAGCTCCTTGGAACAGTGG
mmu-Lbp-R	GAGGTGGGCAGGATCACAAA
mmu-C3-F	AGCTTCAGGGTCCCAGCTAC
mmu-C3-R	CTCTCCAGCCGTAGGACATT
mmu-Ddc F	ATCCCGACAACAGAGTGGAC
mmu-Ddc R	GGACGACCCTCAATGCCATC
mmu-Lpin2 F	TGGAAATGAAAGGCCAGCTCA
mmu-Lpin2 R	GCCTGCTCCTCCTTCCTATTG
mmu-Acot1 F	AGTGCTGATTCAAGGGCTGG
mmu-Acot1 R	TTCTCGCAGCTGGATTGAAC
mmu-Zfpm1 F	CCCTGTGCAGGAACCAGTAG
mmu-Zfpm1 R	ACCAGATCCCGCAGTCTTTG
mmu-Cdh1-F	ATGTCCTGGGCAGAGTGAGA
mmu-Cdh1-R	TGGAGCTTTAGATGCCGCTT
mmu-GAPDH-F	ATGACTCCACTCACGGCAAA
mmu-GAPDH-R	CGGCCTCACCCCATTTGATG

Supplemental Table S2. List of antibodies used for western blot analysis.

Antibody	Isotype	Dilution	Source	Class
Primary antibody				
Nrf2	Rabbit	1:500	Bioss (Beijing, China)	polyclonal
Keap1	Rabbit	1:1000	proteintech (Wuhan, China)	polyclonal
Hmox1	Rabbit	1:1000	proteintech (Wuhan, China)	polyclonal
β-actin	Mouse	1:1000	proteintech (Wuhan, China)	monoclonal
Second antibody				
IRDye 800CW	Goat anti-rabbit	1:10,000	LI-COR (USA)	
IRDye 680RD	Goat anti-mouse	1:10,000	LI-COR (USA)	
