# Supplementary figures and images for: Vitamin U Attenuates Acute Aflatoxin B1-Induced Liver Injury in Mice: Biochemical, Histological and Transcriptomic Evidence
Source: Vet Sci. 2026 Jun 26;13(7):621. doi: 10.3390/vetsci13070621 (PMC13431570; doi:10.3390/vetsci13070621)

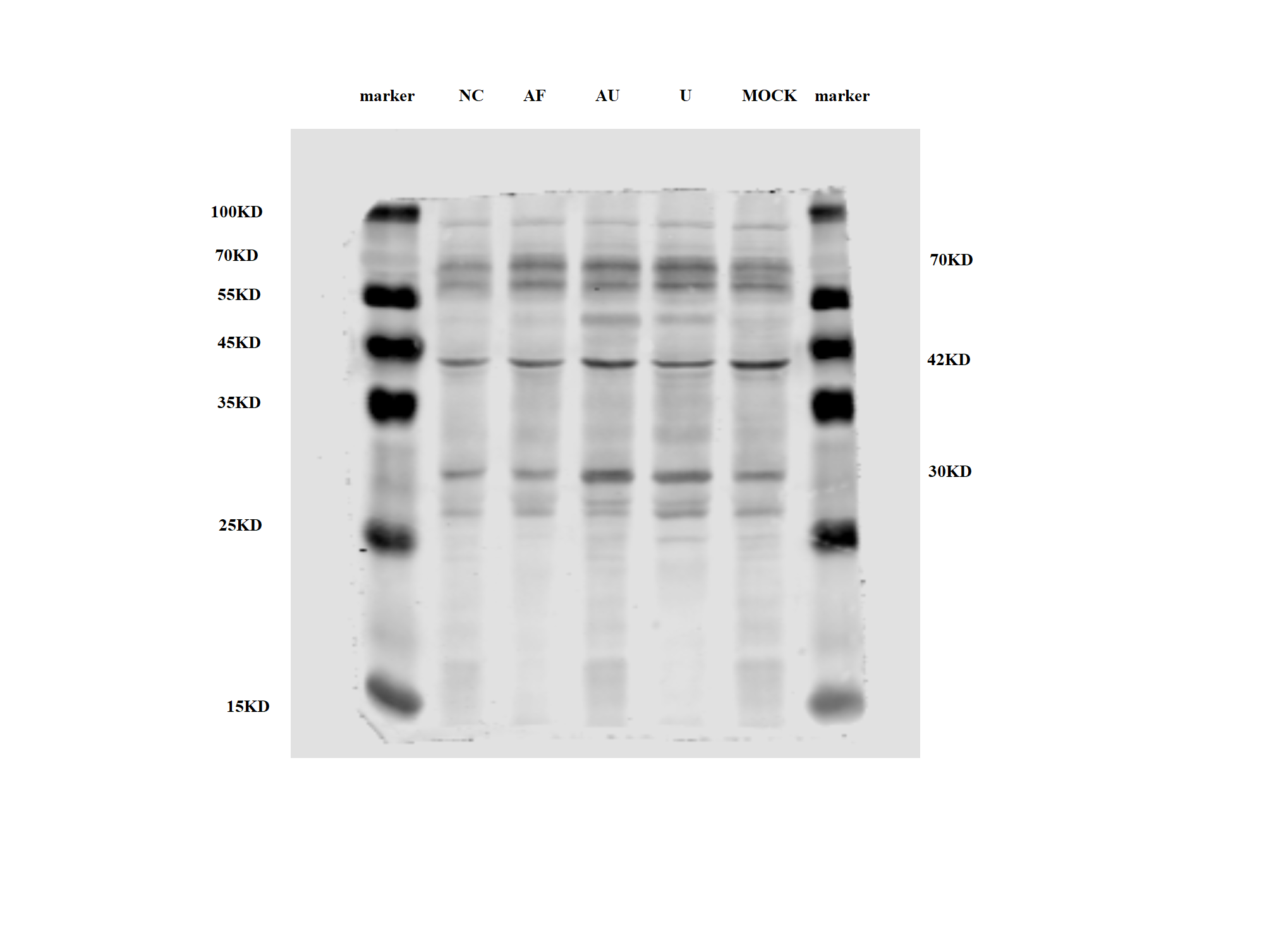

Supplement: Supplementary file 1 [file vetsci-13-00621-s001.zip › vetsci-4317631-supplementary File S1/Keap1 Hmox1/1 beta-actin Keap1 Hmox1 for Figure 6A-marked.tif]

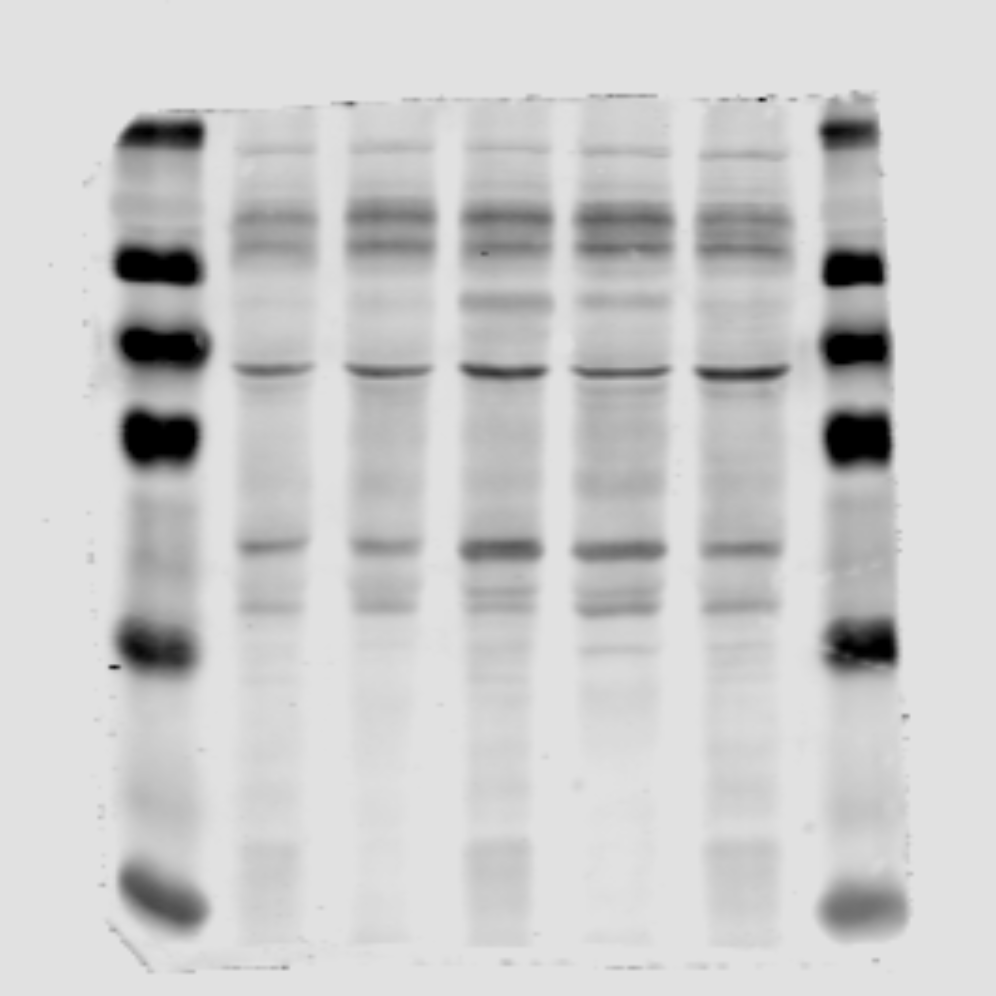

Supplement: Supplementary file 1 [file vetsci-13-00621-s001.zip › vetsci-4317631-supplementary File S1/Keap1 Hmox1/1 beta-actin Keap1 Hmox1 for Figure 6A.tif]

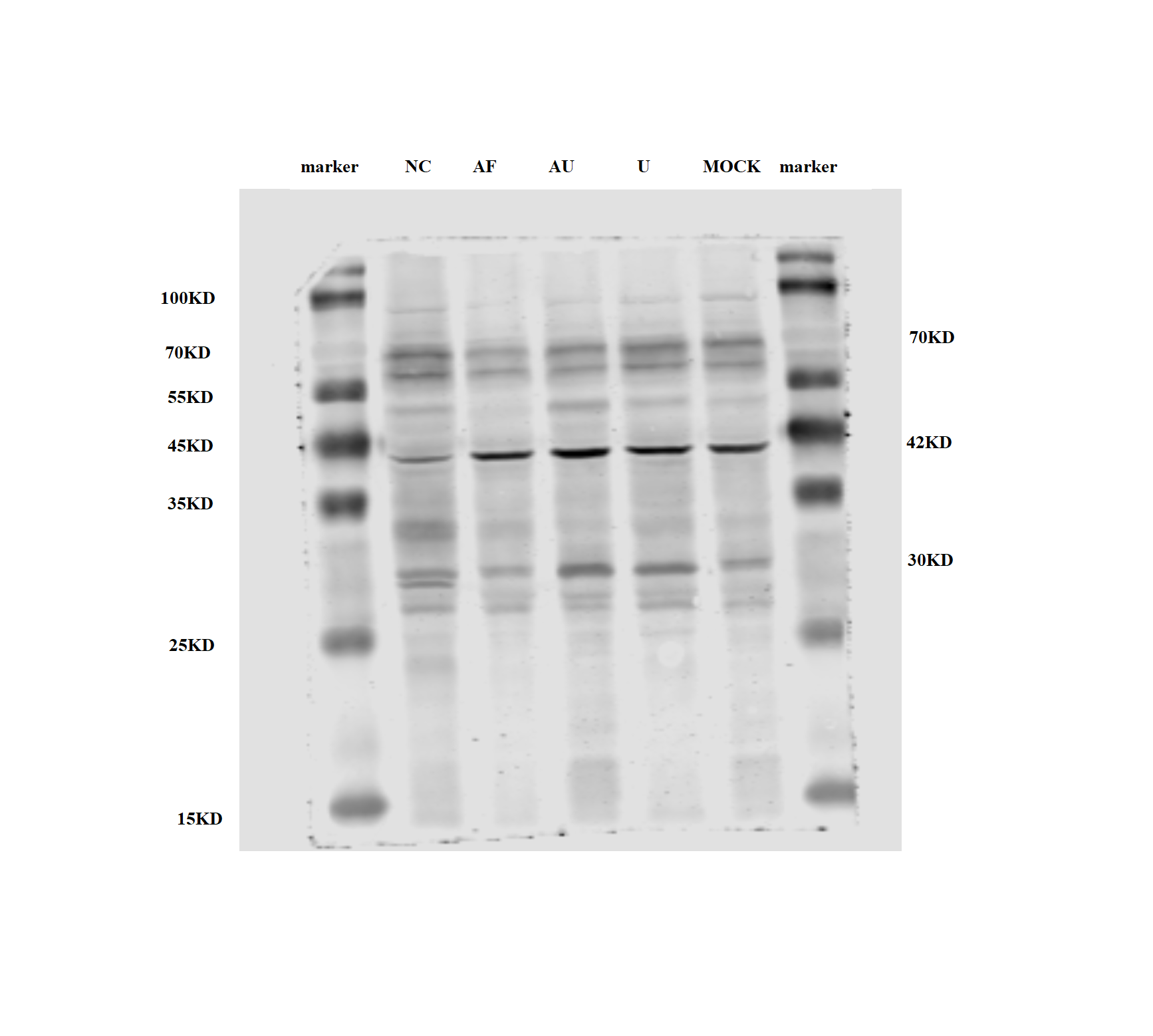

Supplement: Supplementary file 1 [file vetsci-13-00621-s001.zip › vetsci-4317631-supplementary File S1/Keap1 Hmox1/2 beta-actin Keap1 Hmox1 for Figure 6A-marked.tif]

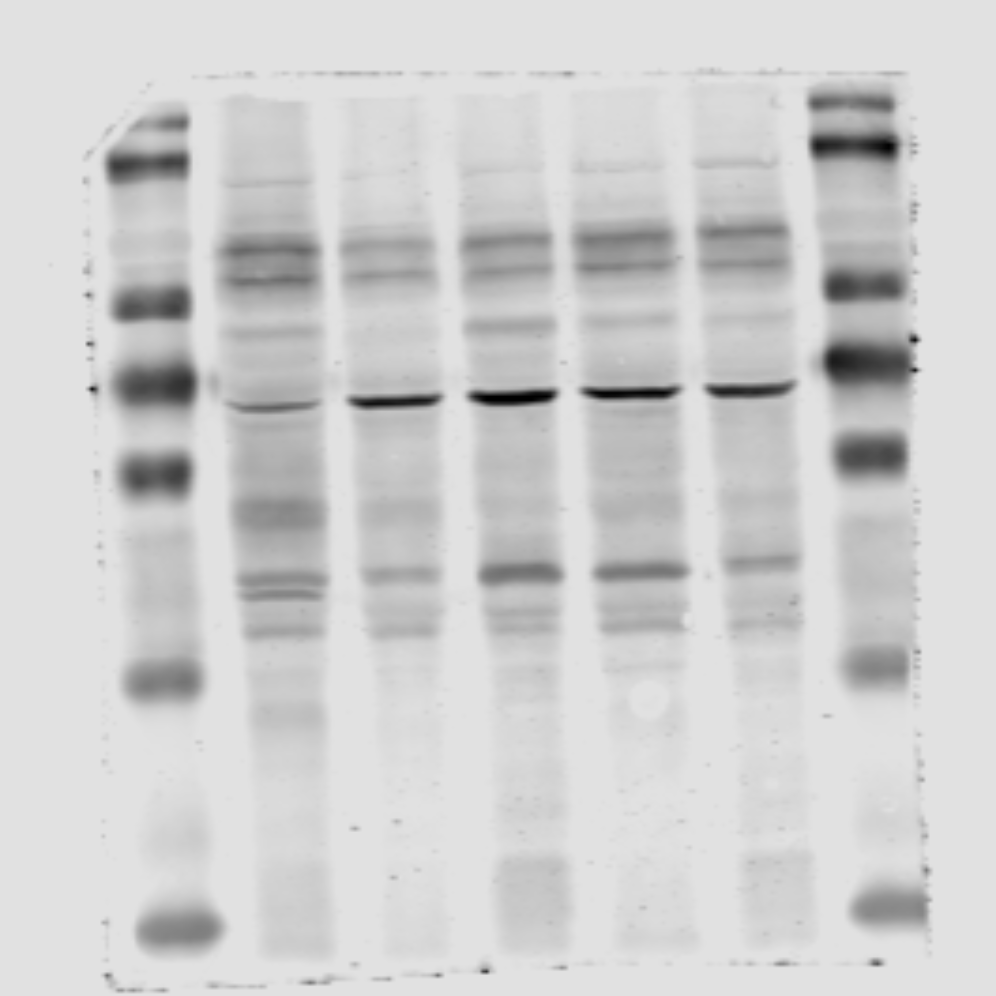

Supplement: Supplementary file 1 [file vetsci-13-00621-s001.zip › vetsci-4317631-supplementary File S1/Keap1 Hmox1/2 beta-actin Keap1 Hmox1 for Figure 6A.tif]

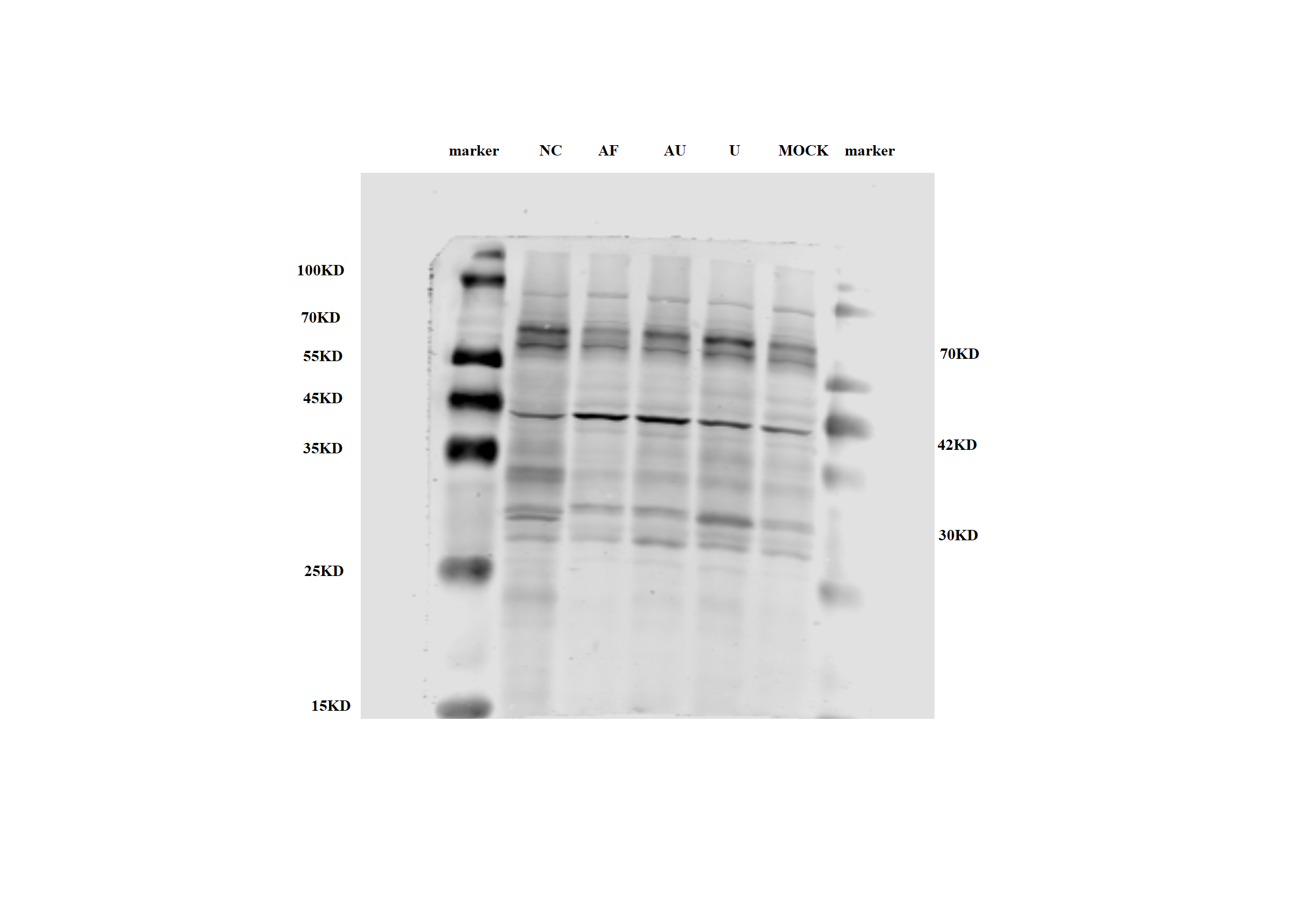

Supplement: Supplementary file 1 [file vetsci-13-00621-s001.zip › vetsci-4317631-supplementary File S1/Keap1 Hmox1/3 beta-actin Keap1 Hmox1 for Figure 6A-marked.tif]

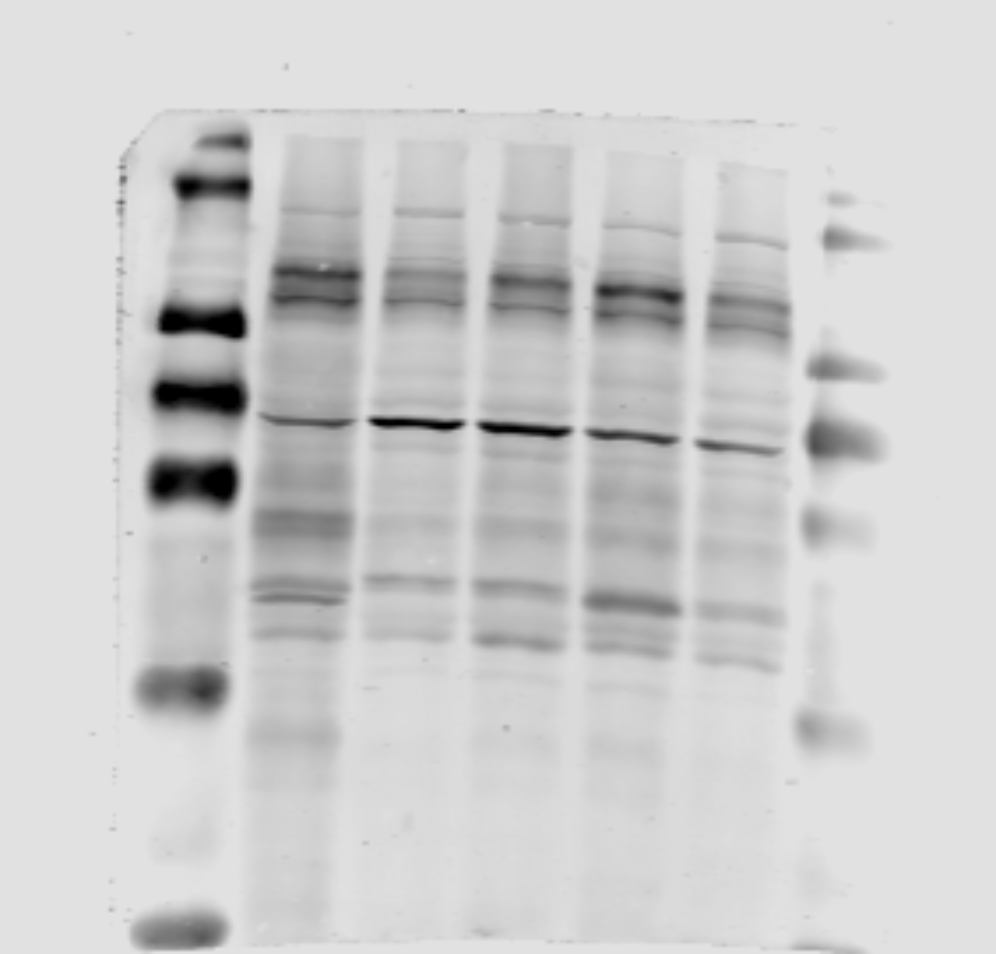

Supplement: Supplementary file 1 [file vetsci-13-00621-s001.zip › vetsci-4317631-supplementary File S1/Keap1 Hmox1/3 beta-actin Keap1 Hmox1 for Figure 6A.tif]

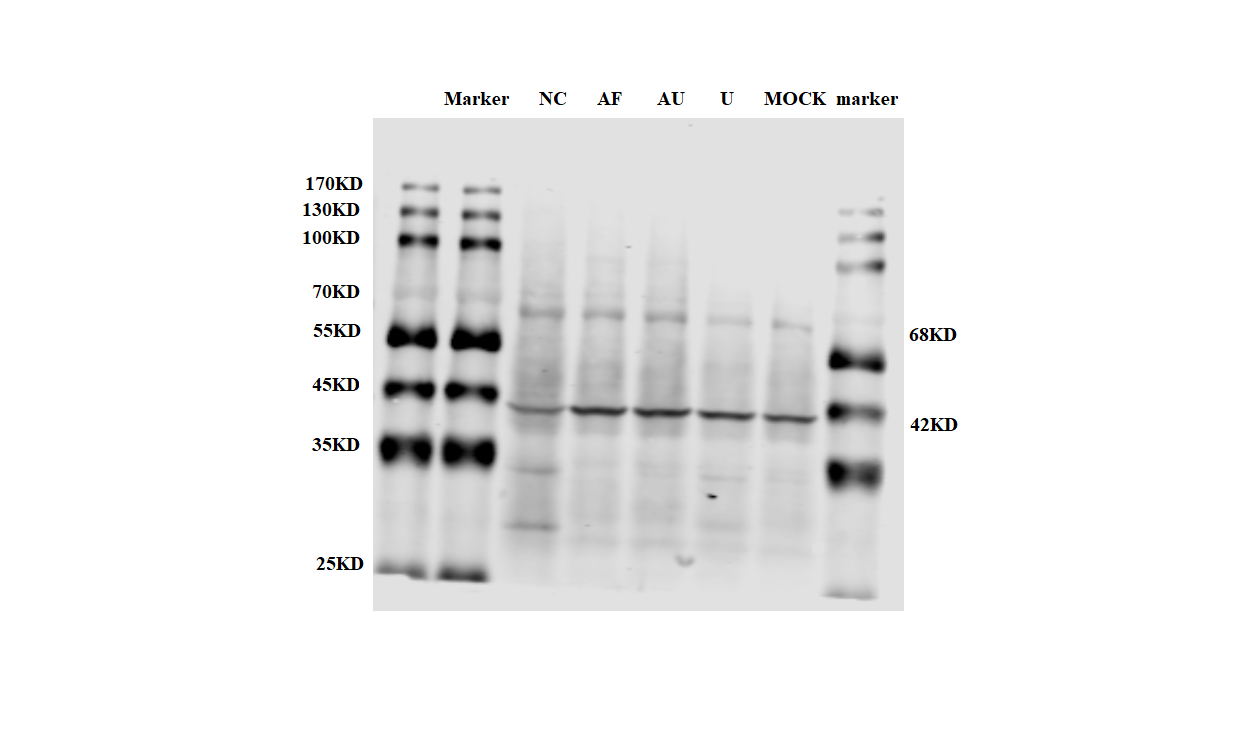

Supplement: Supplementary file 1 [file vetsci-13-00621-s001.zip › vetsci-4317631-supplementary File S1/Nrf2/1 beta-actin Nrf2 for Figure 6A-marked.tif]

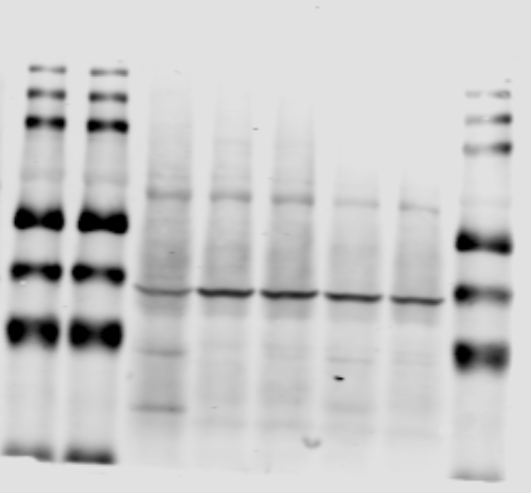

Supplement: Supplementary file 1 [file vetsci-13-00621-s001.zip › vetsci-4317631-supplementary File S1/Nrf2/1 beta-actin Nrf2 for Figure 6A.tif]

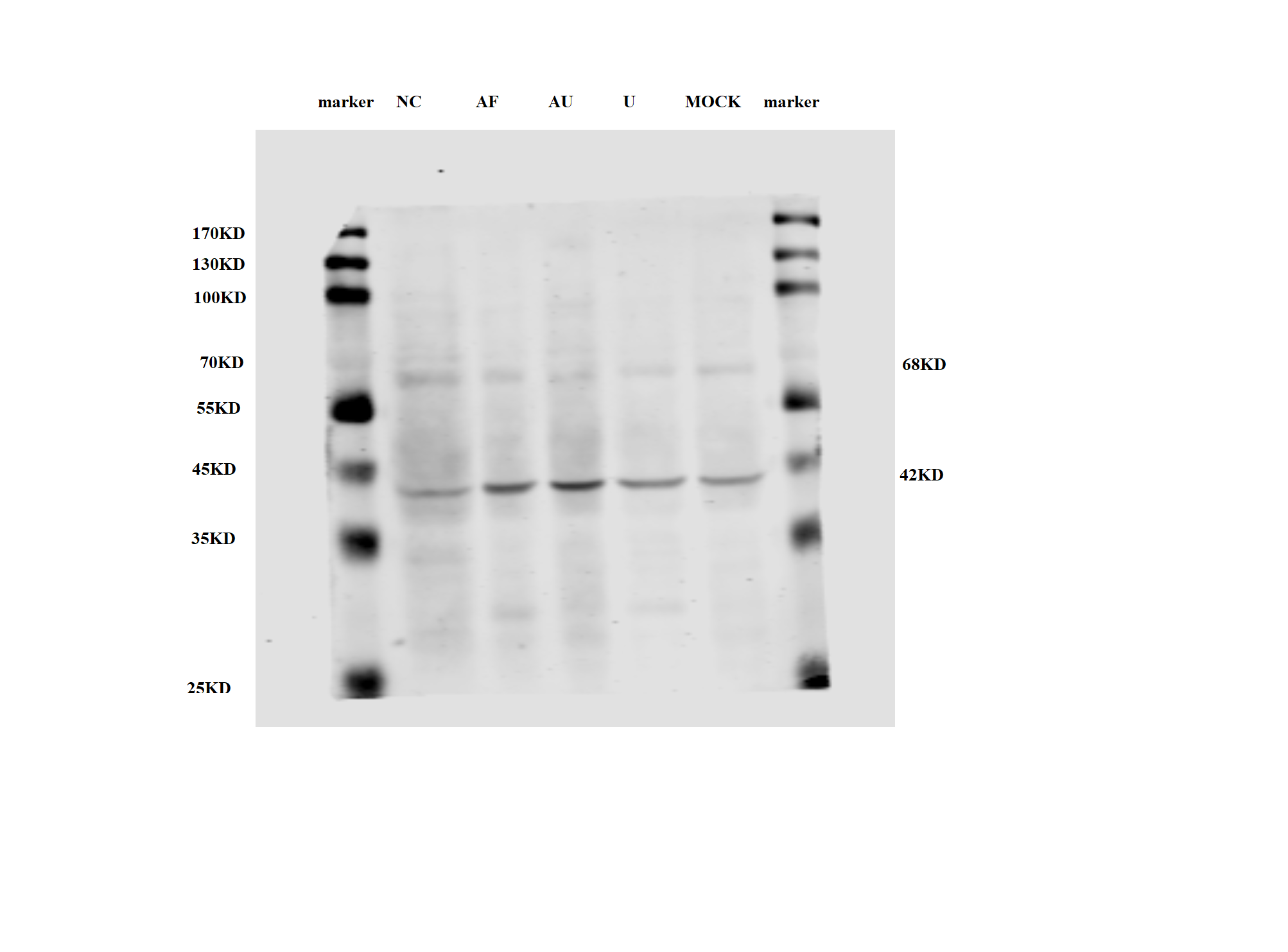

Supplement: Supplementary file 1 [file vetsci-13-00621-s001.zip › vetsci-4317631-supplementary File S1/Nrf2/2 beta-actin Nrf2 for Figure 6A-marked.tif]

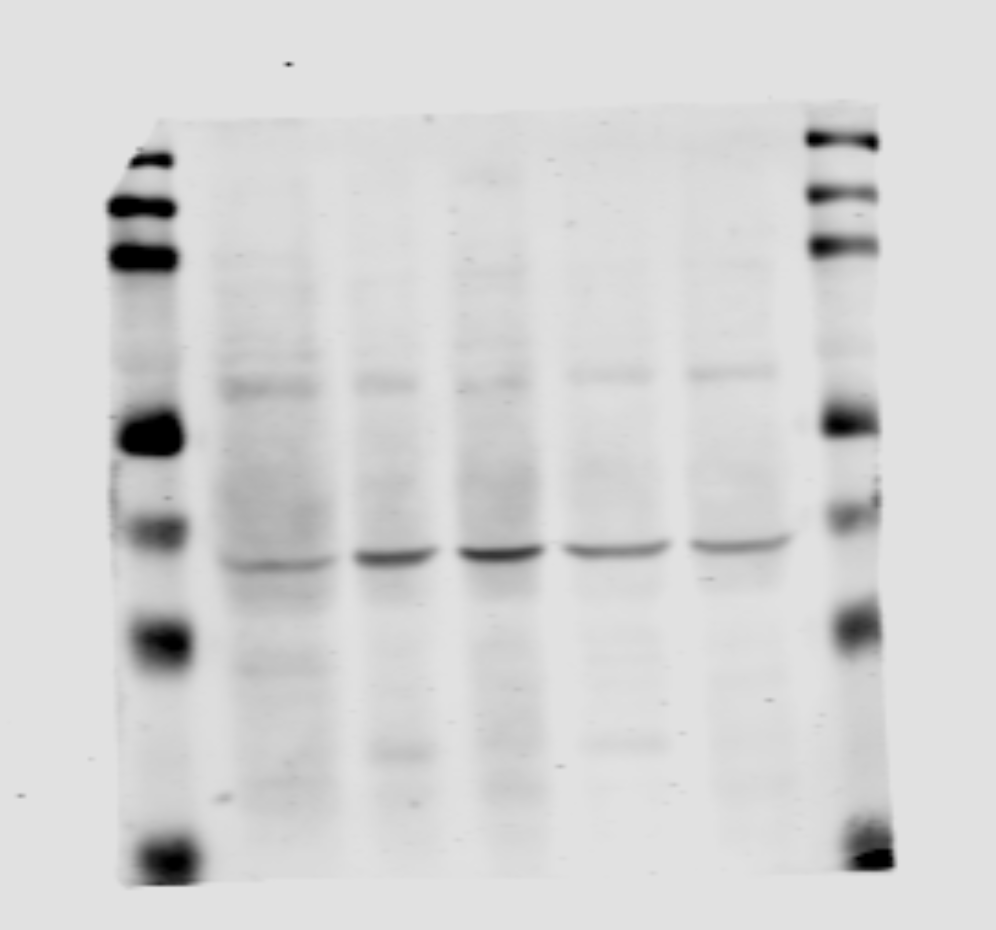

Supplement: Supplementary file 1 [file vetsci-13-00621-s001.zip › vetsci-4317631-supplementary File S1/Nrf2/2 beta-actin Nrf2 for Figure 6A.tif]

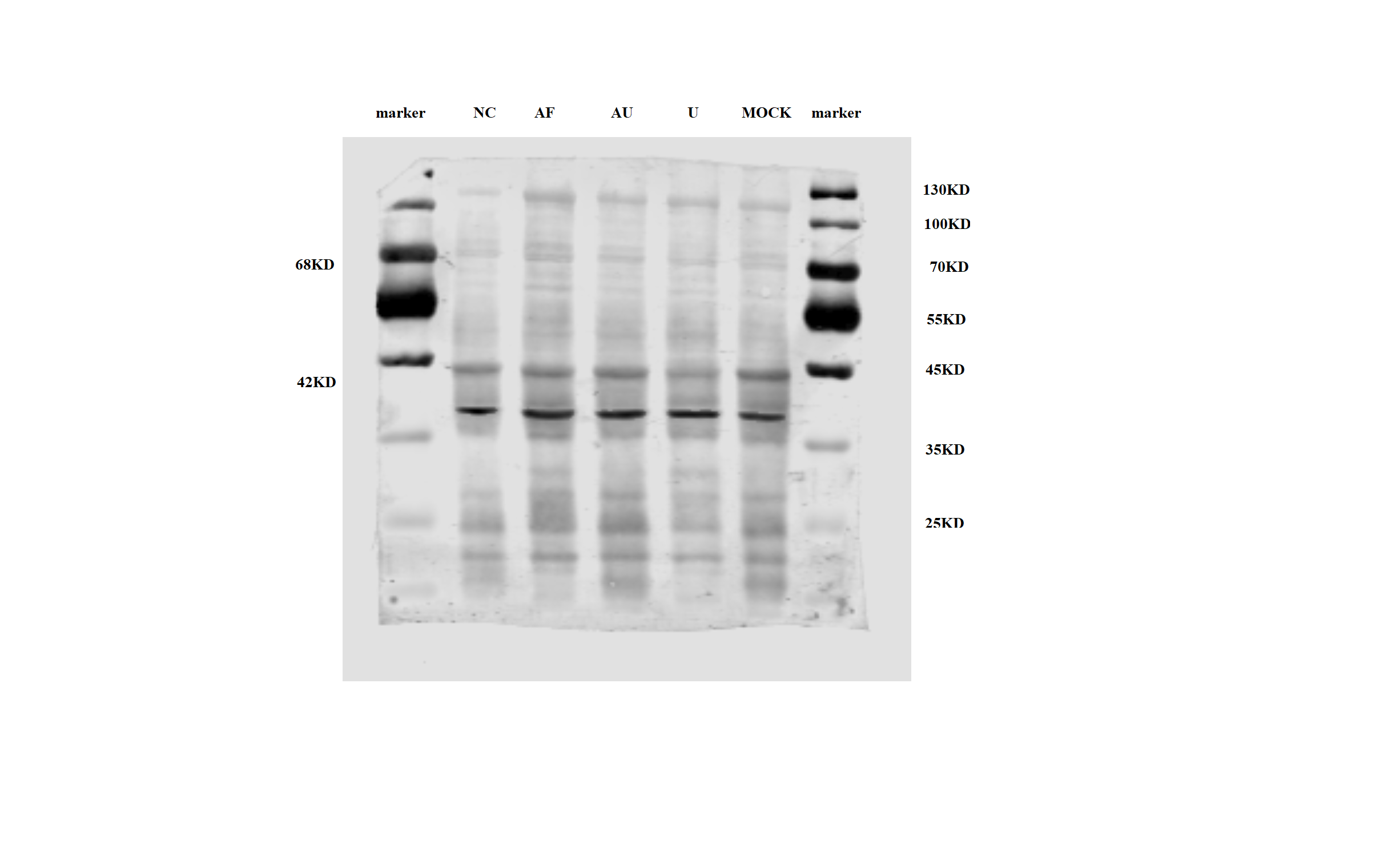

Supplement: Supplementary file 1 [file vetsci-13-00621-s001.zip › vetsci-4317631-supplementary File S1/Nrf2/3 beta-actin Nrf2 for Figure 6A-marked.tif]

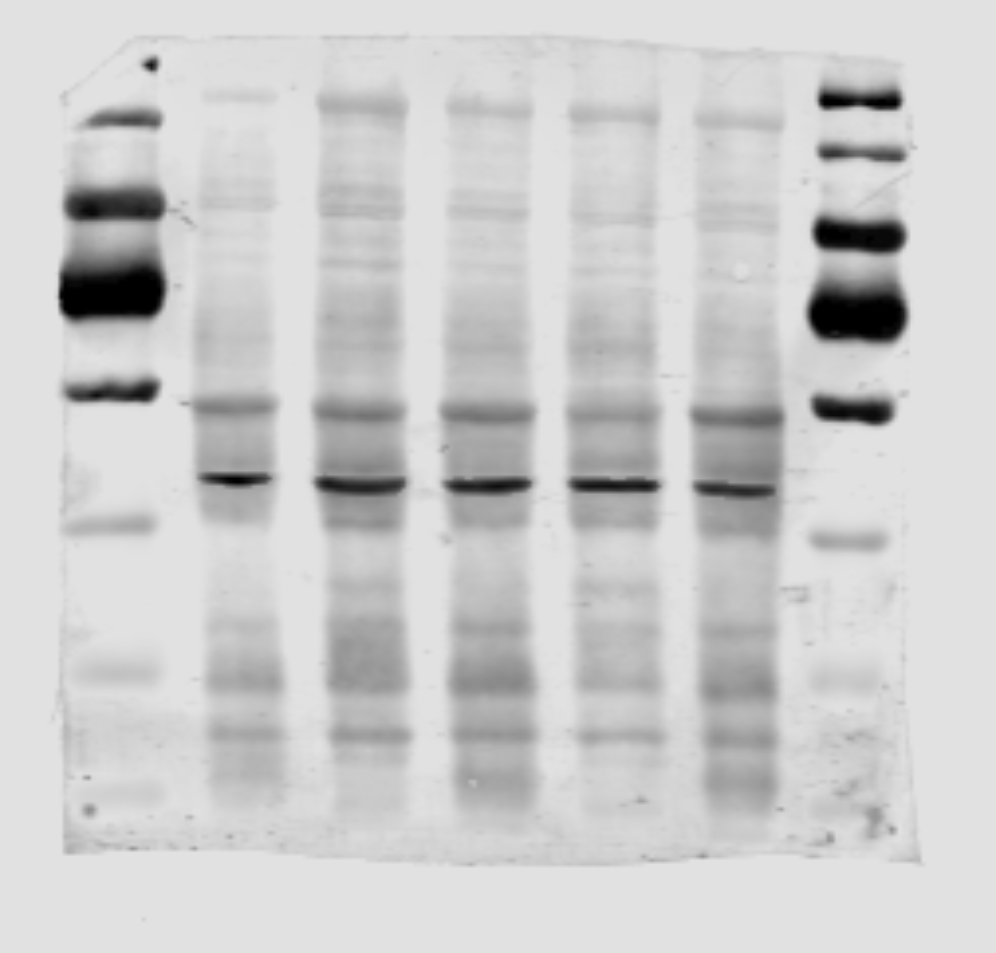

Supplement: Supplementary file 1 [file vetsci-13-00621-s001.zip › vetsci-4317631-supplementary File S1/Nrf2/3 beta-actin Nrf2 for Figure 6A.tif]
